# Conformational Modulation of Tissue Transglutaminase via Active Site Thiol Alkylating Agents: Size Does Not Matter

**DOI:** 10.3390/biom14040496

**Published:** 2024-04-19

**Authors:** Pauline Navals, Alana M. M. Rangaswamy, Petr Kasyanchyk, Maxim V. Berezovski, Jeffrey W. Keillor

**Affiliations:** Department of Chemistry and Biomolecular Sciences, University of Ottawa, Ottawa, ON K1N 6N5, Canada; pnavals@uottawa.ca (P.N.); arang029@uottawa.ca (A.M.M.R.); pkasy042@uottawa.ca (P.K.); maxim.berezovski@uottawa.ca (M.V.B.)

**Keywords:** transglutaminase 2, transglutaminase inhibitors, targeted covalent inhibitors, irreversible inhibitors, G-protein, conformational modulation

## Abstract

TG2 is a unique member of the transglutaminase family as it undergoes a dramatic conformational change, allowing its mutually exclusive function as either a cross-linking enzyme or a G-protein. The enzyme’s dysregulated activity has been implicated in a variety of pathologies (e.g., celiac disease, fibrosis, cancer), leading to the development of a wide range of inhibitors. Our group has primarily focused on the development of peptidomimetic targeted covalent inhibitors, the nature and size of which were thought to be important features to abolish TG2’s conformational dynamism and ultimately inhibit both its activities. However, we recently demonstrated that the enzyme was unable to bind guanosine triphosphate (GTP) when catalytically inactivated by small molecule inhibitors. In this study, we designed a library of models targeting covalent inhibitors of progressively smaller sizes (15 to 4 atoms in length). We evaluated their ability to inactivate TG2 by measuring their respective kinetic parameters *k*_inact_ and *K*_I_. Their impact on the enzyme’s ability to bind GTP was then evaluated and subsequently correlated to the conformational state of the enzyme, as determined via native PAGE and capillary electrophoresis. All irreversible inhibitors evaluated herein locked TG2 in its open conformation and precluded GTP binding. Therefore, we conclude that steric bulk and structural complexity are not necessary factors to consider when designing TG2 inhibitors to abolish G-protein activity.

## 1. Introduction

**Figure 1:**
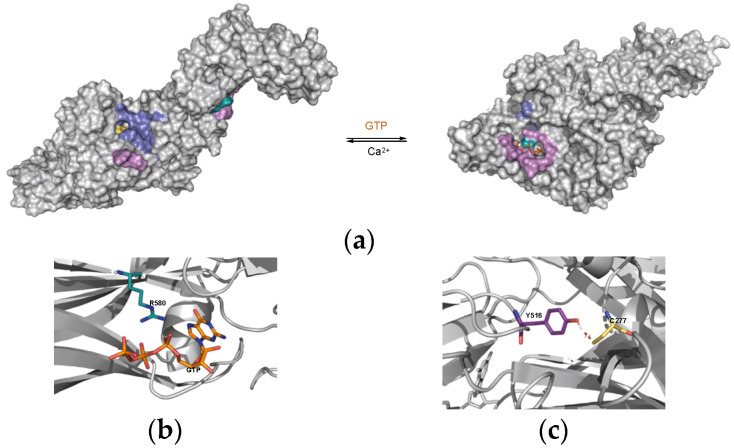
Structures of major TG2 conformations. (**a**) TG2 in its calcium-stabilized conformation (open and linear, with a fully formed catalytic binding site in blue and the active Cys277 residue in yellow, PDB ID: 2Q3Z) and its GTP-bound conformation (closed and compact, with a GTP-binding site formed in purple, PDB ID: 6A8P). (**b**) Focus on the proximity of Arg580 (cyan) and GTP (orange) in the GTP-bound conformation of TG2. (**c**) Focus on the interaction between Tyr516 (deep purple) and Cys277 (yellow) in the GTP-bound conformation of TG2.

Mammalian transglutaminases (TGs) are multifunctional calcium-dependent enzymes that are primarily known for their ability to cross-link proteins between Gln and Lys residues through the formation of an *N*^ε^(γ-glutaminyl)lysine bond [1,2]. Human tissue transglutaminase (TG2), a ubiquitous and unique member of the TG family, catalyzes the formation of this covalent bond by mediating an acyl transfer mechanism through its active site cysteine residue (Cys277, Figure 1a, yellow) [1,3,4]. Structurally, TG2 consists of four distinctive domains: an *N*-terminal β-sheet domain, a catalytic domain, and two *C*-terminal β-barrel domains [5,6,7]. Unlike other TGs, TG2 plays a second major role as a G-protein [8], which is associated with a dramatic conformational change, allowing it to bind and hydrolyze GTP, and it is known in this role as G_h_α [6,9,10,11,12]. In the extracellular matrix, TG2 adopts a linear, open conformation that is stabilized by binding calcium. In this conformation, the transamidase active site’s catalytic triad is formed (Cys277, His335, and Asp358), and substrates can bind and be crosslinked or hydrolyzed (Figure 1a, blue) [6,10]. Intracellularly, where calcium levels are low, TG2 adopts a more compact conformation in which its two *C*-terminal β-barrels fold over its *N*-terminal domain, rendering the catalytic residues inaccessible to the substrate and subsequently forming a GTP-binding site (Figure 1, purple) [6,11].

The allosteric mechanisms governing this conformational change have been studied, and evidence has been provided to underline the importance of two residues in the transition between the two states. Firstly, Arg580 (Figure 1b R580 in cyan) has been identified as a switch residue, strongly destabilizing the closed and catalytically inactive conformation in the absence of direct interaction with GTP (Figure 1c, in orange) [10,13]. Secondly, Tyr516 has been proposed to stabilize the closed conformation via the formation of a non-canonical “hydrogen bond” with the thiolate group of the catalytic Cys277 residue (Figure 1c, Y516 in deep purple) [5,14]. Finally, the mutation of the Cys277 into an alanine residue (C277A) led to a complete destabilization of the closed conformation in the presence or absence of GTP [5].

TG2′s ubiquity in tissues has led to its implication in a variety of pathologies. Its acyltransferase activity (through its open conformation) has been shown to play a significant role in celiac disease [14,15] and pulmonary and liver fibrosis [16,17], as well as in a variety of cancers [18,19,20,21]. Its role as a G-protein (in its closed conformation) has been implicated in the cell signalling pathways that are critical for the survival of cancer stem cells and for metastasis [22,23,24]. Consequently, considerable work has been conducted in the development of potent TG2 inhibitors to block these signalling pathways [22,25,26]

Inhibitors of TG2 are diverse, ranging from small molecules to peptides, including both reversible inhibitors and irreversible targeted covalent inhibitors (TCIs) [22,27,28,29]. Our group has predominantly focused on the development of peptidomimetic TCIs designed to react with the catalytic Cys277 of TG2, locking the enzyme in its open conformation and preventing it from folding to form its GTP-binding site [30,31,32,33]. Historically, we believed that the steric bulk imparted by a large peptidic or peptidomimetic inhibitor scaffold was necessary to prevent the conformational change that would allow GTP binding and subsequent function as a G-protein. However, we recently published the results of a SAR study that explored the structural requirements of the small TCI molecules of TG2 [34] and were surprised at their ability to inhibit both its transamidase and GTP binding activities. These results led us to question our previous assumption as to the importance of the size of TCIs in abolishing GTP binding. Herein, we disclose the design, synthesis, and inhibitory evaluation of a new library of increasingly smaller TG2 TCIs to determine if a minimal length exists at which an inhibitor will no longer abolish GTP binding while still blocking its transamidase activity.

## 2. Materials and Methods

### 2.1. Protein Expression and Kinetic Assay

Recombinant TG2 was expressed and purified from *E. coli* as previously described [35]. TG2 activity was determined according to a previously published colorimetric activity assay using the chromogenic substrate Cbz-Glu(γ-p-nitrophenyl ester)-Gly-OH, also known as AL5 [36]. To determine the irreversible inhibition parameters *k*_inact_ and *K*_I_, enzymatic assays for each inhibitor were run in triplicate under Kitz and Wilson conditions [37] ([AL5] substrate = 100 μM). Buffered solutions of 50 mM of 3-(4-morpholino)propanesulfonic acid (MOPS) (pH 6.91), 15.5 mM CaCl_2_, 100 μM AL5, and various concentrations of inhibitor (from 10 µM to 10 mM, depending on the inhibitor) were prepared in a 96-well polystyrene microplate with a final volume of 200 μL at 25 °C. AL5 and inhibitor stocks were prepared in DMSO, ensuring that the final concentration of this co-solvent did not exceed 6% *v*/*v*. If necessary, the working stocks of each inhibitor were diluted with water to maintain less than 6% *v*/*v* DMSO. To initiate the enzymatic reaction, 5 mU mL^−1^ of TG2, or water for the blank, was added to the well, and the formation of the hydrolysis product, *p*-nitrophenolate, followed at 405 nm for 20 min using a BioTek Synergy 4 plate reader (Agilent, Santa Clara, CA, USA). The first-order rate constants of inactivation (*k*_obs_) were obtained by fitting the absorbance versus time data via non-linear regression to mono-exponential Equation (1) using GraphPad Prism 7 software.

(1) $${Abs}_{t}={Abs}_{max}(1-e^{-k_{\mathrm{obs}}t})$$

The rate constants were then fitted via non-linear regression to a saturation kinetics model using Equation (2).

(2) $$k_{\mathrm{obs}}=\frac{k_{inact}\cdot [I]}{\left[I\right]+K_{I}\cdot \alpha }$$

To correct for the competition with the assay substrate AL5, the inhibitor concentrations were divided by α, which equals 1 + [S]/*K*_M_, where *K*_M_ = 10 μM. The *k*_inact_/*K*_I_ efficiency ratio was calculated either from the *k*_inact_ and *K*_I_ values derived from non-linear regression to Equation (2) or via the linear regression of the lowest concentration data point.

### 2.2. GTP Binding Assay

GTP binding experiments were performed following an adapted and previously described protocol [34]. TG2 (20 μg) was incubated at 25 °C for 30 min with or without an irreversible inhibitor (each at a concentration of 2 × *K*_I_) with 15 mM CaCl_2_ in 100 mM MOPS (pH = 6.91). Iodoacetamide was used at a concentration of 0.52 µM, at 6.9-fold its K_I_ value but equimolar with TG2, in order to ensure the stoichiometric labelling of the enzyme. The buffer was then exchanged via dialysis to 100 mM MOPS (pH = 7.0), 1 mM EGTA, and 5 mM MgCl_2_ to remove calcium using a 14 kDa molecular weight cut-off membrane cuvette (purchased from Millipore-Sigma, Oakville, ON, Canada). The fluorescent, nonhydrolyzable GTP analogue BODIPY GTP-γ-S (purchased from Invitrogen, Waltham, MA, USA), for which its fluorescence increases when bound to the protein, was then added at a final concentration of 0.5 μM. Fluorescence was then measured on a microplate reader after 10 min of incubation (Ex/Em: 490/520 nm).

### 2.3. Native PAGE Assay

Native PAGE experiments were performed using a protocol adapted from those previously described [5,6]. TG2 (2.5 µM) was incubated at 25 °C for 30 min with or without irreversible inhibitor (each at a concentration of 2 × *K*_I_) with 15 mM CaCl_2_ in 100 mM MOPS (pH = 6.91). Iodoacetamide was used at a concentration of 2.5 µM, at 33-fold its *K*_I_ value, but it was equimolar with TG2 in order to ensure the stoichiometric labelling of the enzyme. Three controls were performed: (1) The uninhibited TG2 was analysed in the presence of calcium (100 mM MOPS + 15 mM CaCl_2_) and in the absence of GTP; (2) the uninhibited TG2 was analysed in the absence of both calcium and GTP; (3) uninhibited TG2 was analysed in the presence of GTP (100 mM MOPS, 1 mM EGTA, and 1 mM GTP) but in the absence of calcium. A 20 μL aliquot (or 1.5 µg of TG2) of each solution was diluted with 20 μL of native Laemmli 2× buffer (62.5 mM Tris-HCl (pH 6.8), 40% glycerol, 0.01% bromophenol blue); then, 20 μL of each solution was loaded onto a 4–20% Mini-PROTEAN precast gel (Bio-Rad, Hercules, CA, USA). The protein species were separated at 125 V for 90 min using a running buffer without SDS (25 mM Tris, 192 mM glycine, pH 8.3) at 4 °C. The gel was stained using a Coomassie brilliant blue R-250 solution and de-stained with 10% acetic acid and 25% methanol in water.

### 2.4. Capillary Electrophoresis Assay

For kinetic capillary electrophoresis (KCE) experiments, TG2 (2.5 µM) was incubated at 25 °C for 30 min with an irreversible inhibitor (each at a concentration of 25.0 µM) with 15 mM CaCl_2_ in 100 mM MOPS (pH 6.91). After incubation, the samples were buffer-exchanged with 20 mM tris-acetate (pH 7.21) solution using 30 kDa molecular cut-off polyethersulfone membrane centrifugal filters (VWR) at 4 °C and concentrated to a final TG2 concentration of ~25 μM. For the control experiments, a stock TG2 solution was directly buffer exchanged in the same conditions, concentrated to ~25 μM, and subjected to KCE analysis. Capillary electrophoresis analysis was performed using the Beckman Coulter Proteome Lab PA 800 capillary electrophoresis system with a photodiode array (PDA) detector. A fused silica capillary with a 75 μm I.D., 375 µm O.D., and 50 cm total length and measuring 40 cm relative to the detection window length (Beckman Coulter, Brea, CA, USA) was used for all runs. Before the first use, the new capillary was conditioned via washing for 30 min with a 0.1 M NaOH solution, followed by a 30 min wash with the 0.1 M HCl solution and a 30 min wash with double-distilled water. Prior to each sample run, the capillary was rinsed with 0.1 M NaOH for 0.5 min at 15 psi pressure, distilled water for 0.5 min at 15 psi, 0.1 M HCl for 0.5 min at 15 psi, and a separation buffer for 1 min at 20 psi. Samples were introduced into the capillary using hydrodynamic injection for 5 s at a pressure of 0.5 psi. All separations were performed within a 600 V/cm electric field in the normal polarity mode (positive voltage applied to the electrode in the inlet). The capillary temperature was maintained at 15.0 °C. The PDA signal was monitored at 190 nm. The separation of the inhibited TG2 samples was carried out in a 20 mM tris-acetate buffer solution (pH 7.21). For the control experiments, the uninhibited TG2 sample was subjected to separation in four buffers: (1) 20 mM tris-acetate (pH 7.21); (2) 20 mM tris-acetate (pH 7.21) with 200 μM CaCl_2_; (3) 20 mM tris-acetate (pH 7.21) with 200 μM MgCl_2_; and (4) 20 mM tris-acetate (pH 7.21) with 200 µM MgCl_2_ and 100 µM Na-GTP.

### 2.5. Synthesis

Solvents and reagents were all purchased from commercial sources and directly used without further purification. Compounds **7** and **8**, also known as *N*-methylacrylamide and acrylamide, respectively, and iodoacetamide were also purchased from commercial sources. All ^1^H-NMR and ^13^C-NMR spectra were recorded on 300 and 400 MHz Bruker spectrometers, respectively, and all chemical shifts are reported in ppm referenced to the deuterated solvent peak. Each spectrum can be found in the Supplementary Material. High-resolution mass spectra (HRMS) were obtained with a quadrupole time-of-flight (QTOF) analyser and electrospray ionization (ESI). Thin-layer chromatography (TLC) was performed using aluminum-backed silica plates and visualized using UV light unless specified differently. The purity of all compounds was assessed via reverse phase HPLC, and purity was reported to be above 95% (see Supplementary Material for HPLC traces).

## 3. Results and Discussion

### 3.1. Design

**Figure 2:**
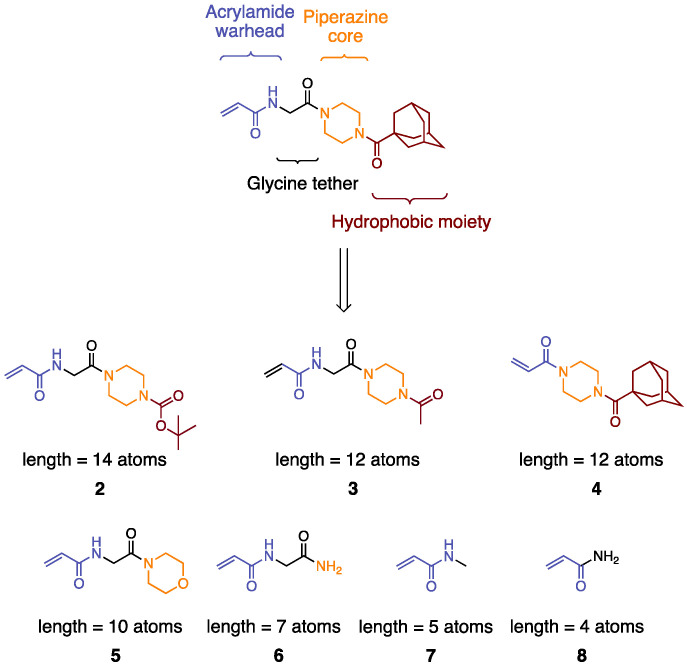
Structure of **1** and the subsequently smaller inhibitors designed via truncation.

Like most covalent inhibitors of its generation, inhibitor **1** (aka **EB-2-16**) is composed of four distinctive moieties that all contribute to its affinity for the TG2 transamidase catalytic site [34,38]. First, it contains an acrylamide warhead (Figure 2, in blue) that reacts with the Cys277 of the catalytic site to form a thioether bond in irreversible inhibition. Directly linked to it by an amide bond, a glycine tether can be found, which has been determined to have the optimal length for potency and provides some conformational flexibility (Figure 2, in black) [34]. The terminal adamantyl group (Ad, Figure 2 in dark red) has been described as providing high affinity for the hydrophobic “D-site” of the substrate binding site of TG2 [34,38]. Finally, these units are bridged in **1** via a piperazine spacer that positions the hydrophobic moiety appropriately (Figure 2, in orange) [30].

To determine the minimal size at which an inhibitor would no longer prevent GTP binding, we designed a library of molecules through systematic modification or the complete removal of most of the structural elements of **1** while conserving the acrylamide warhead. The inhibitors deriving from this design strategy are shown in Figure 2, and they are ordered according to the ‘length’ of the molecule, which is defined as the number of atoms from the acrylamide electrophilic beta carbon. Briefly, we imagined that the hydrophobic moiety of **1** could be reduced in size from Ad to Boc (**2**) or acetyl (**3**), decreasing the overall length from 16 atoms to 14 or 12, respectively. A third compound, also 12 atoms in length, could also be generated by alternatively removing the glycine tether, as in compound **4**. The hydrophobic moiety could be entirely removed from the original structure, leading to compound **5**, where the piperazine core is replaced by a morpholine, reducing the length to 10 atoms. This would then be followed by complete removal of the piperazine core, resulting in **6**, at 7 atoms in length. The next inhibitor would be generated by replacing the core, tether, and hydrophobic unit of **1** altogether with a simple methyl group (**7**, 5 linear atoms). Finally, the smallest inhibitor of our library would comprise the acrylamide warhead alone as a primary carboxamide, commonly known as acrylamide (**8**).

### 3.2. Synthesis

**Scheme 1:**
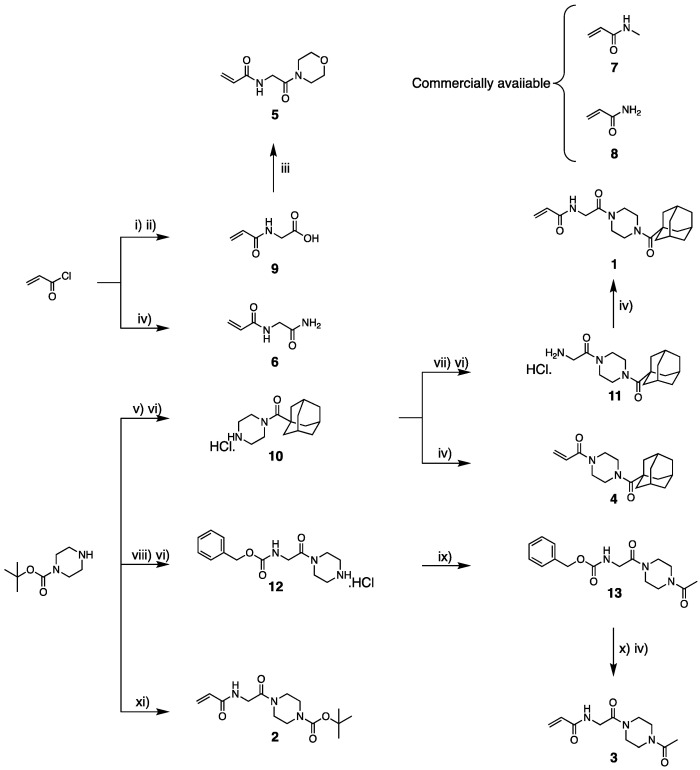
Synthetic routes of small molecule inhibitors. Reagents and conditions: (i) H-Gly-O*t*Bu. HCl, HCl (1 eq), DIPEA (2 eq) in dry DCM under N_2_ at 0 °C, and then 10 min at R.T. (ii) Neat TFA in excess 1 h at R.T. (iii) Morpholine (1.3 eq), NMI (3.5 eq), and TCHF (1.2 eq) in ACN under N_2_ overnight at R.T. (iv) Acryloyl chloride (1.1 eq) and DIPEA (2 eq) in dry DCM at 0 °C and then 1 h at R.T. under N_2_. (v) Adamantyl chloride (1.1 eq) and DIPEA (1 eq) in DCM 1 h at R.T. (vi) HCl (4 M) in dioxane, excess, 1 h at R.T. (vii) Boc-glycine (1.1 eq), DIPEA (2 eq) 1 h at R.T. (viii) Cbz-Gly-OH (1.1 eq), HATU (1.6 eq), DIPEA (2 eq) in DCM overnight at rt. (ix) TEA (3 eq), acetyl chloride (1.5 eq) in dry DCM under N_2_ at 0 °C then 6 h at R.T. (x) Pd/C 10 wt% in dry MeOH under N_2_ and then NaBH_4_ added slowly (2 eq); then, 3 h at R.T. (xi) Nine (1.1 eq) DMAP (0.1 eq) and DCC (1.1 eq) in dry DCM under N_2_ overnight at R.T.

Due to the structural similarity of all these small inhibitors, synthetic schemes for their preparation were based on diversification either from the piperazine core, for the inhibitors conserving it, or from the acrylamide warhead for the smallest inhibitors, as shown in Scheme 1.

Compound **1** was first prepared via the functionalization of Boc-piperazine with adamantanecarbonyl chloride, followed by Boc deprotection, leading to amine **10**. This amine was then coupled with Boc-glycine and subsequent Boc deprotection led to the hydrochloride salt **11**. A final coupling with acryloyl chloride gave **1**. From intermediate amine **10**, compound **4** was easily accessible through direct condensation with acryloyl chloride. Compound **3** was prepared through the orthogonal functionalization of Boc-piperazine with Cbz-glycine, as none of the coupling conditions we explored ever allowed conversion between acetyl-piperazine and Boc-glycine. The removal of its Boc-protecting group led to intermediate **12**. The acetylation of **12** produced the Cbz-protected intermediate **13**, which was then deprotected with sodium borohydride and directly coupled to acryloyl chloride, resulting in the final compound **3**. The synthesis of **2** was achieved via the direct coupling of Boc-piperazine and intermediate **9**, commonly known as acryloylglycine. The latter was also used to synthesize compound **5** via condensation with morpholine. Finally, the coupling of commercially available glycinamide with acryloyl chloride allowed the formation of inhibitor **6**. Inhibitors **7** and **8** were obtained from commercial sources. Detailed synthetic procedures, ^1^H and ^13^C NMR spectra, HRMS values, and HPLC traces of all inhibitors can be found in the Supplementary Materials.

### 3.3. Kinetic Evaluation of Inhibition

**Figure 3:**
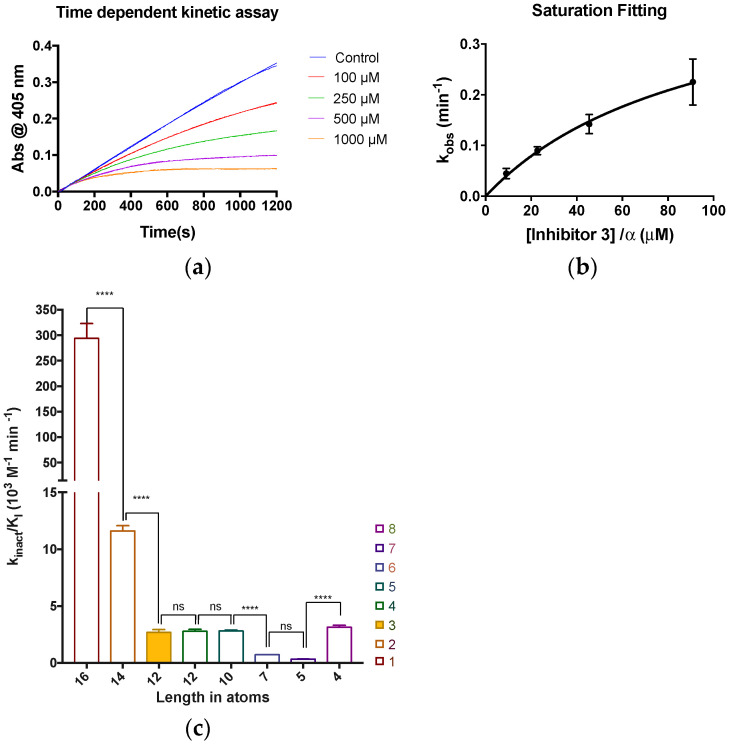
(**a**) Time-dependent absorbance curves of substrate AL5 providing the observed rate constants (*k*_obs_) of the inhibition of human TG2 by inhibitor 3. (**b**) Hyperbolic fitting of the different *k*_obs_ rate constants vs. the concentration of inhibitor 3 to a saturation kinetics model. (**c**) *k*_inact_/*K*_I_ efficiency ratio vs. length of inhibitor, with statistical significance shown for the difference of each value from the preceding value (**** *p* value < 0.0001; ns = not significant).

The goal of this study was not to design potent and efficient new inhibitors but rather to evaluate the minimum size of inhibitor required to abolish TG2′s ability to bind GTP. However, it was first necessary to confirm the activity of the inhibitors against transamidation activity, which was achieved using our well-established colorimetric kinetic assay [36]. Briefly, rate constants for the time-dependent inactivation of TG2 (*k*_obs_) were measured under Kitz and Wilson conditions by monitoring the enzymatic hydrolysis of chromogenic substrate AL5 in the presence of various concentrations of the inhibitor (Figure 3a). These rate constants were then fitted to a hyperbolic equation consistent with saturation kinetics (all fits can be found in the SI), providing the inhibition parameters *k*_inact_ and *K*_I_ (Figure 3b). The inhibition constants (*K*_I_), inactivation rate constants (*k*_inact_), and the overall efficiency ratio *k*_inact_/*K*_I_ of all inhibitors are presented in Table 1. In addition, the *k*_inact_/*K*_I_ parameters derived from the simple linear regression of *k*_obs_ values measured at the lowest concentrations are provided to allow comparison with the ratios calculated from the independently determined parameters.

We have previously evaluated the impact that structural modifications of this class of inhibitors can have on their kinetic parameters of inhibition [30,34] and were thus unsurprised to observe a wide range of activity from this series. However, interestingly, the overall efficiencies, as indicated by the *k*_inact_/*K*_I_ ratio, seem to be correlated to inhibitor length (Figure 3c). Decreasing the length to 14 (**2**) and 12 atoms (**3**) significantly decreases overall efficiency from (294 ± 29) × 10^3^ M^−1^ min^−1^ to (11.6 ± 0.5) and (2.69 ± 0.25) × 10^3^ M^−1^ min^−1^, respectively. Similar efficiencies were observed for compounds **3**, **4**, and **5** (10–12 atoms), with *k*_inact_/*K*_I_ values of (2.69 ± 0.25), (2.79 ± 0.17), and (2.81 ± 0.08) × 10^3^ M^−1^ min^−1^, which are not significantly different from each other. A further decrease in efficiency was observed when reducing the length to seven and five atoms, corresponding to compounds **6** and **7**, which showed *k*_inact_/*K*_I_ values of (0.35 ± 0.02) and (0.730 ± 0.001) × 10^3^ M^−1^ min^−1^. These values are significantly different from those of the previous range but not from each other. Finally, and somewhat surprisingly considering the previous trend, a slight but significant increase in the overall efficiency was observed for the smallest inhibitor, **8**, with a *k*_inact_/*K*_I_ value of (3.15 ± 0.17) × 10^3^ M^−1^ min^−1^.

### 3.4. GTP Binding Evaluation

**Figure 4:**
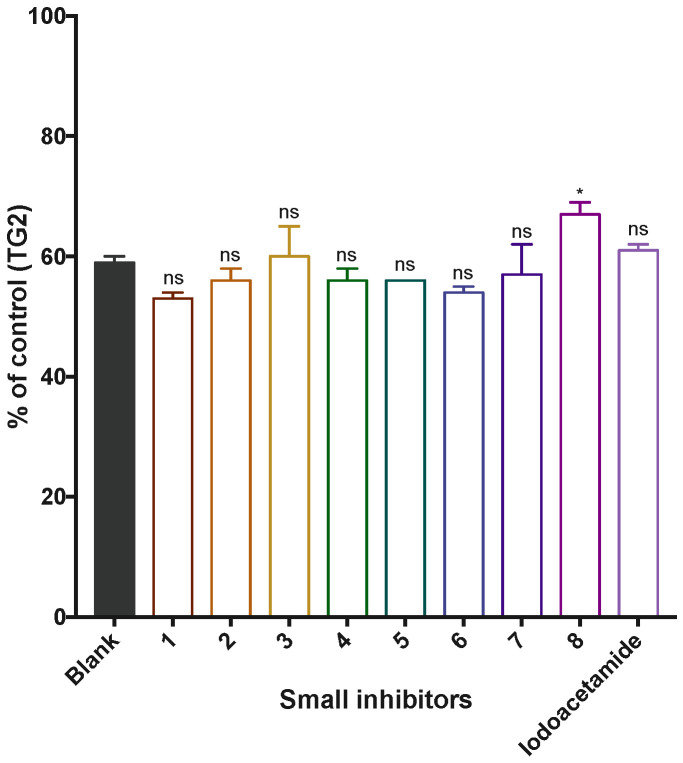
Fluorescence from the GTP binding assay after the incubation of TG2 with small molecule inhibitors. Values are shown as the percentage of fluorescence observed for the positive control, in which TG2 was not inhibited prior to GTP binding. The blank represents the signal of the fluorescent GTP analogue used in the binding assay in the absence of TG2 (ns = not significant; * *p* value < 0.05, relative to the positive control of TG2 + BODIPY-GTP).

We then evaluated the capacity of each inhibitor to permit or preclude GTP binding by TG2. To do so, TG2 was first inactivated via incubation with each inhibitor at a concentration corresponding to twice their respective *K*_I_ value in a calcium-rich buffer. The buffer was then exchanged via dialysis to remove the calcium. A fluorescent GTP analogue (BODIPY-GTP) was then added to the solution at a constant concentration, and relative fluorescence (RFU) was recorded (λEx/Em: 490/520 nm). Finally, these RFU values were compared to a control representing the maximum signal of the bound GTP analogue in the absence of an inhibitor and to a blank representing the intrinsic signal of the fluorescent GTP analogue used in the absence of TG2. The results are presented in Figure 4, where it is obvious that all these small inhibitors prevented GTP binding, regardless of their length, size, or structure. All inhibitors reduced the fluorescence signal to the same level as the blank, which represents the intrinsic fluorescence measured for the unbound GTP analogue, in the absence of TG2. This clearly indicates that none of the small inhibitors allow GTP binding. Surprisingly, these results, and more specifically the result for compound **8**, are in opposition to what was previously observed for iodoacetamide, known as the smallest irreversible inhibitor of TG2 [22,30]. Consequently, we decided to re-examine the GTP binding with iodoacetamide. Importantly, we incubated TG2 in the presence of an equimolar concentration of iodoacetamide (0.52 µM) in order to allow for the complete labelling of TG2. (We note here that the iodoacetamide concentration used in the previous study [30], corresponding to 2-fold the *K*_I_ for iodoacetamide (namely at 150 nM [39]), would not have allowed for the stoichiometric labelling of the enzyme.) After incubation in the presence of an equimolar concentration of iodoacetamide, we observed in the current study that even the smallest TG2 irreversible inhibitor does not allow for GTP to bind with the enzyme (Figure 4).

### 3.5. Conformational Analysis by Native PAGE and Capillary Electrophoresis

Because TG2 is capable of large conformational dynamics, it was also imperative to probe the potential relationship between the suppression of GTP binding observed for all inhibitors and the enzyme’s conformational state. To do so, two distinctive conformational analyses were performed on uninhibited and inhibited TG2. We first conducted a qualitative native polyacrylamide gel electrophoresis analysis (nPAGE), which has been previously used very effectively to differentiate between the two main conformational states of TG2 [5,6,38,40]. Previous studies have clearly shown that the open conformation of TG2 migrates more slowly on a native PAGE gel and can be separated from the faster migrating closed form [5,6].

**Figure 5:**
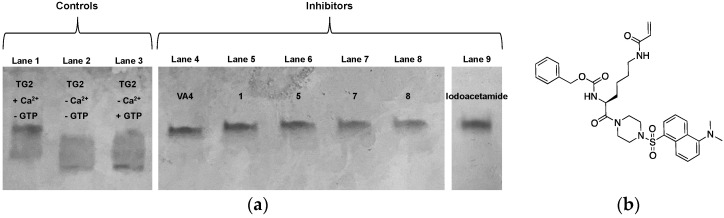
(**a**) Conformational analysis by native PAGE. A contrast-adjusted gel is shown here. The original, unaltered image may be found in the Supplementary Material. (**b**) Structure of inhibitor **VA4**.

For this experiment, three controls were performed: (1) uninhibited TG2 was analysed in the presence of calcium and in the absence of GTP, serving as a positive control for its ‘open’ extended conformation; (2) uninhibited TG2 was analysed in the absence of both calcium and GTP, representing TG2 in its free forms; (3) uninhibited TG2 was analysed in the presence of GTP but in the absence of calcium, ultimately representing its ‘closed’ compact conformation. Inhibitor 1 and three of our smallest inhibitors (**5**, **7**, and **8**) were then evaluated for their effect on TG2 conformation. In addition, a large peptidomimetic inhibitor previously developed by our group, **VA4** (Figure 5b), was also evaluated, as it was previously shown to abolish TG2′s ability to bind GTP [30]. Finally, iodoacetamide was again included in this analysis, as the smallest known inhibitor of TG2.

As shown in Figure 5a, although the presence of calcium establishes a conformational equilibrium that favours the open conformation (Figure 5a, lane 1), all inhibitors tested lock TG2 exclusively in an open extended conformation. Similarly, the incubation of TG2 with iodoacetamide appears to alter the conformational distribution from that of unmodified TG2 (Figure 5a, lane 2), which is consistent with our observation herein that it prevents GTP binding. These results suggest that after the reaction with TG2 and after the formation of the covalent thioether bond, TG2 conformational dynamism seems to be abolished upon alkylation with an inhibitor as small as a three-atom chain.

**Figure 6:**
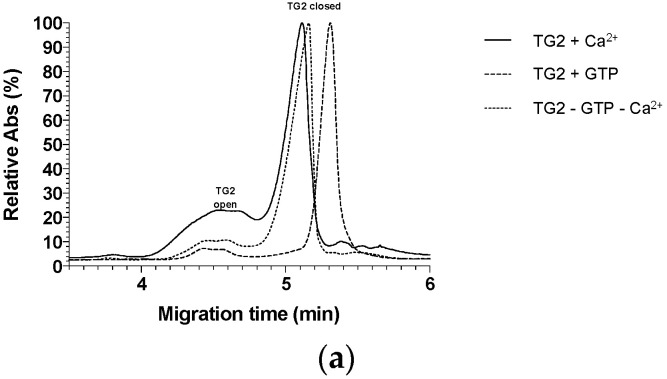

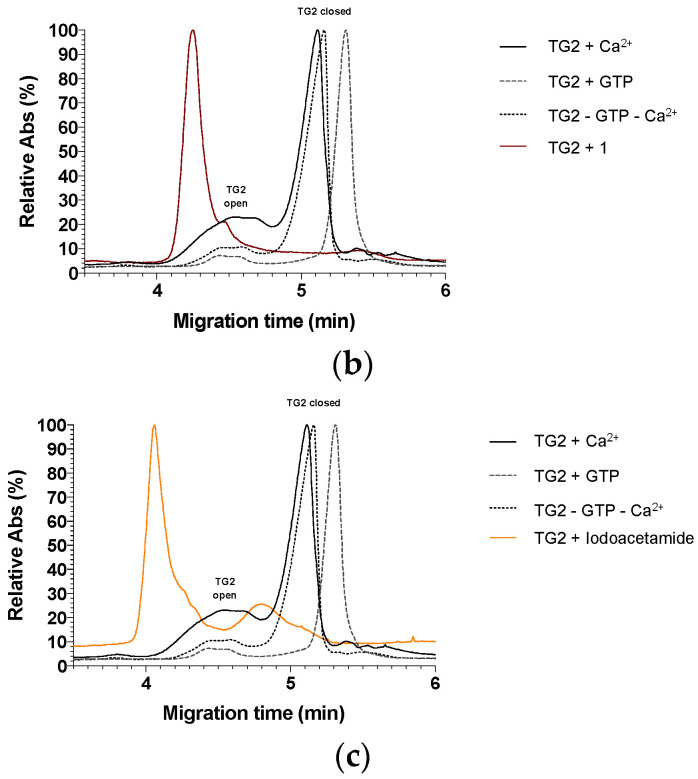
(**a**) Electropherograms comparing the three controls (focused on 3.5 to 6.0 min). (**b**) Electropherogram comparing TG2 incubated with inhibitor **1** and the three controls (focused on 3.5 to 6.0 min). (**c**) Electropherogram comparing TG2 incubated with the inhibitor iodoacetamide and the three controls (focused on 3.5 to 6.0 min).

In addition, we performed a series of capillary electrophoresis analyses on uninhibited and inhibited TG2 to visualize any disparities in migration time between all the possible conformations. We have previously used this method to differentiate between TG2 conformations and showed that the closed conformation migrates more slowly than the open one [41,42]. The comparative analysis of all electropherograms (Figure S2) and the full electropherograms can be found in the Supplementary Materials. The same three controls were used (Figure 6a): (1) TG2 inhibited in the presence of Ca^2+^, representing TG2 in equilibrium between its main conformations; (2) uninhibited TG2 in the presence of GTP and absence of Ca^2+^; and (3) uninhibited TG2 with no Ca^2+^ nor GTP present. Regardless of the environment of the running buffer, the migration times of the open conformation do not vary from one control to another. We always observed the same broad peak spanning the 4.0 to 4.8 min range, reaching maximum absorbance at 4.6 min (Figure 6b). Interestingly, the observed migration times for the closed conformation shift slightly from 5.12 min (Controls 1 and 3) to 5.31 min when GTP is present (Control 2), suggesting that TG2 might adopt a slightly more compact conformation when stabilized with the nucleoside triphosphate, resulting in a slightly slower migration on the capillary.

As expected from our nPAGE results, the results show that TG2 is mostly in its linear, open conformation once alkylated by any of our inhibitors (Supplementary Materials Figure S2). We observe that the shape and intensity of the peak of the open conformation is highly impacted once TG2 is alkylated and a slight migration time shift is always observed. For example, in the case of TG2 incubated with inhibitor **1**, the large broad peak (4.0 to 4.8 min) observed in each control analysis becomes sharper, presents a stronger relative absorbance intensity (from 23% relative absorbance to 100%), and migrates slightly faster with a migration time at 4.25 min (Figure 6b). Even more interestingly, while **VA4** and inhibitor **1** seem to completely abolish TG2 conformational dynamism by locking it in the open conformation, smaller inhibitors (**5**, **7**, **8**, and iodoacetamide) seem to allow for some degree of conformational dynamism (Supplementary Materials Figure S2). In each case, a broad peak of a slower migration time of approximately 4.85 min appears in between the open and closed conformation (Figure 6c). Although we cannot provide any insight regarding the structure of an intermediate conformation within the scope of this study, it is tempting to speculate that after modification by smaller inhibitors, TG2 may adopt a conformation that may resemble the ‘partially closed’ form in which another transglutaminase enzyme has been crystallised [43].

## 4. Conclusions

We sought to determine if a threshold inhibitor size exists below which TG2 could be catalytically inactivated but still bind GTP. To do this, we designed and synthesized a panel of eight small molecule TCIs through a systematic decrease in their length. We used a direct and continuous chromogenic assay to measure their respective irreversible inhibition parameters *k*_inact_ and *K*_I_. The *k*_inact_/*K*_I_ ratios of these inhibitors, representing their overall efficiency, varied systematically as a function of inhibitor size. Intuitively, this is unsurprising; as steric bulk was trimmed away from the optimized inhibitor **1**, the smaller inhibitors were less and less efficient. However, it is important to note that all inhibitors were still capable of leading to the complete irreversible inhibition of TG2. We then demonstrated the capacity of these very small inhibitors to abolish the enzyme’s ability to bind GTP and limit the conformational dynamism of TG2. Notably, we showed that irreversible inhibitors that alkylate the enzyme with a chain as short as four heavy atoms can also abolish GTP binding. This is in complete agreement with the findings of Begg et al. as to the importance of the electronic stabilization of the closed conformation by Cys277 [5]. This provides an important implication for the design of inhibitors, knowing that all agents that alkylate Cys277 will also abrogate GTP binding and, therefore, the G-protein function of TG2. Furthermore, it is important to remember that chemical probes designed to react with TG2 inside living cells will also disrupt GTP binding and G-protein activity in that context. This suggests that if TG2 inhibitors are to be used as cellular research tools to *only* block its cross-linking activity, considerations should be carried out to ensure the inhibitors remain cell impermeable. Otherwise, our results suggest that all cell-permeable inhibitors of TG2 are likely to block both the cross-linking and G-protein activity of this multifunctional protein.

## Figures and Tables

**Table 1:** Kinetic evaluation results of all small inhibitors.

Comp	Length (Heavy Atoms)	*K*_I_ (µM)	*k*_inact_ (min^−1^)	*k*_inact_/*K*_I_ (10^3^ M^−1^ min^−1^)	*k*_inact_/*K*_I_ (10^3^ M^−1^ min^−1^) from Linear Regression
**1**	16	3.2 ± 1.0	1.30 ± 0.20	412 ±142	294 ± 29
**2**	14	129 ± 53	1.80 ± 0.60	14.0 ± 7.0	11.6 ± 0.5
**3**	12	96 ± 17	0.46 ± 0.05	4.70 ± 1.00	2.69 ± 0.25
**4**	12	118 ± 59	0.52 ± 0.16	4.40 ± 2.30	2.79 ± 0.17
**5**	10	487 ± 161	1.80 ± 0.50	3.60 ± 1.50	2.81 ± 0.08
**6**	7	842 ± 25	0.42 ± 0.01	0.50 ± 0.02	0.35 ± 0.02
**7**	5	6980 ± 5050	5.30 ± 3.70	0.764 ± 0.760	0.730 ± 0.001
**8**	4	148 ± 68	0.70 ± 0.22	4.70 ± 2.60	3.15 ± 0.17
**Iodoacetamide ^a^**	3	0.075 ± 11 ^a^	0.90 ± 0.06 ^a^	12,000 ± 1 ^a^	n.a.

^a^ Previously reported in reference [39].

## Data Availability

Data will be provided upon reasonable request.

## Supplementary Materials

The following supporting information can be downloaded at: https://www.mdpi.com/article/10.3390/biom14040496/s1. Synthesis of compounds **1** to **6** (pp. 2–5); all NMR spectra (pp. 6–9); HPLC data. Table S1: HPLC purity data for synthesized inhibitors **1** to **6** (pp. 10–13); Figure S1: original image of the full-length native PAGE gels. Gel 1: Controls (lanes **1** to **3**) + inhibitor **VA4**, **1**, **5**, **7**, **8**, and iodoacetamide (lanes 4 to 9). Gel 2: Controls (lanes 3 and 4) and iodoacetamide (5 eq and 10 eq, lanes 5 and 6) (p. 14); Figure S2: KCE electropherograms of comparative analysis of all analytes. KCE individual electropherograms of all analytes (pp. 17–20); Kinetic data fitting (pp. 21–23). References [34,44,45,46] are cited in Supplementary Materials.
