# Supplementary material for: Conformational Modulation of Tissue Transglutaminase via Active Site Thiol Alkylating Agents: Size Does Not Matter
Source: Biomolecules. 2024 Apr 19;14(4):496. doi: 10.3390/biom14040496 (PMC11048362; doi:10.3390/biom14040496)
Supplement: Supplementary file 1 [file biomolecules-14-00496-s001.zip › biomolecules-2922236-supplementary.pdf]

Supplementary Material for:

Conformational modulation of tissue transglutaminase by active site thiol alkylating agents: size doesn't matter

Pauline Navals[1], Alana M.M. Rangaswamy[1], Petr Kasyanchyk[1], Maxim V. Berezovski[1], & Jeffrey W. Keillor[1,*]

[1]Department of Chemistry and Biomolecular Sciences,
University of Ottawa, Ottawa, Ontario K1N 6N5 Canada*
Corresponding author: jkeillor@uottawa.ca

Table of contents

1. Synthesis **2**

2. NMR Spectra **6**

3. HPLC data **14**

4. Native PAGE original stained gels **21**

5.Capillary electrophoresis full electropherograms **22**

6. Kinetic data fitting **28**

References **31**

1. Synthesis

N-2-[4-[[1-Adamantanecarbonyl]-1-piperazinyl]-2-oxoebenzyl N-[2-oxo-2-(piperazin-1-yl)ethyl]carbamatethyl] propenamide (1) was synthesized as previously described [34].

***tert*-butyl 4-[2-(prop-2-enamido)acetyl]piperazine-1-carboxylate (2).** Compound **9** (0,15 g, 0.8 mmol, 1.1 eq), DCC (0.17 g, 0.8 mmol, 1.1 eq) and DMAP (0.096 mg, 0.08 mmol, 0.1 eq) were dissolved in ACN at room temperature, under N_2. After a few minutes a white precipitate formed and Boc-piperazine (0.14 g, 0.74 mmol, 1 eq) was added. The reaction was monitored by TLC (DCM/MeOH, 95/5) and stirred at room temperature overnight. Dicyclohexylurea (DCU) was then filtered out and ACN was then evaporated. The resulting yellow oil was then dissolved in AcOEt (5 mL) and was subsequently washed with AcOH 5% (3 ×5 mL), brine (2 ×), saturated $NaHCO_3$ (3 × 5 mL) and brine again (3 × 5 mL) before being dried over anhydrous $Mg1SO_4$, filtered and concentrated. The product was then washed with cold Et_2O, and the resulting white powder was isolated in a 30% yield. **^{1}H-NMR ($CDCl_3$, 300 MHz):** 1.48 (9H, s), 3.45 (6H, m), 3.63 (2H, m), 4.15 (2H, d, J=4.13 Hz), 5.69 (1H, dd, J=3.90 Hz), 6.19 (1H, dd, J=9.00 Hz), 6.32 (1H, dd, J=6.25 Hz), 6.69 (1H, s). **^{13}C-NMR ($CDCl_3$, 101 MHz):** 28.36, 33.97, 41.34, 41.92, 44.29, 80.62, 126.96, 130.32, 148.80, 154.6, 165.6. **HRMS-EI:** $[M]^+$ = calculated 297.1689 found 297.1655.

N-[2-(4-acetylpiperazin-1-yl)-2-oxoethyl]prop-2-enamide (3). Compound **13** (0.19 g, 0.69 mmol, 1 eq) and Pd/C (0.02 g, 10% w) were suspended in freshly dried MeOH (7 mL) under N_2 atmosphere, then $NaBH_4$ (0.05 g, 0.66 mmol, 2 eq) was added portion wise to control the emulsion. The reaction was stirred until completion, about 3 h (TLC DCM/MeOH, 95/5). The reaction was quenched with water and the azeotrope H_2O/MeOH was evaporated followed by filtration through celite of the Pd. Then, the crude product (0.1 g, 0.6 mmol, 1 eq) was directly dissolved in dry DCM (5 mL) under N_2 atmosphere and DIPEA (0.3 mL, 1.8 mmol, 3 eq) was added. The mixture was cooled down to 0°C and acryloyl chloride (0.05 mL, 0.66 mmol, 1.1 eq) was added dropwise. The reaction was stirred at 0°C under N_2 until completion, about 1 h (TLC: DCM/MeOH 98%/2%). The DCM was then evaporated, and the crude oil was directly subjected to normal phase flash chromatography (DCM/MeOH, 0 to 10% MeOH) followed by reverse phase chromatography (H_2O + 0.01% TFA/ACN, 10 to 70% ACN). The desired product was isolated in a 5.5% overall yield. **^{1}H-NMR (D_2O, 300 MHz):** 2.13 (3H, s), 3.62 (8H, m, J=4.17 Hz), 4.21 (2H, s), 5.78 (1H, dd, J=3.82 Hz), 6.21 (1H, dd, J=6.12 Hz), 6.33 (1H, q, J=9.13 Hz). **^{13}C-NMR (D_2O, 101 MHz)**: 20.34, 40.95, 41.08, 41.22,

41.85, 43.87, 44.16, 45.45, 127.98, 129.36, 168.89, 172.81. **HRMS-ESI** $[MNa]^+$ = calculated 262.1170 found 262.1168.

Adamantane-1-carbonylpiperazin-1-yl-prop-2-en-1-one (4). Compound **10** (0.125 g, 0.44 mmol, 1 eq) was suspended in DCM (2 mL) under N_2 and DIPEA (0.230 mL, 1.32 mmol, 3 eq) was added. To the now homogenous mixture was added acryloyl chloride (0.039 mL, 0.48 mmol, 1.1 eq), dropwise. The mixture was stirred at room temperature until completion (about 30 min), as determined by TLC (MeOH/DCM, 2% / 98%, visualized with ninhydrin). The solvent was then evaporated, and the residue was dissolved in EtOAc. This organic layer was then washed with AcOH 5% (3 × 10 mL), brine (1 × 10 mL), saturated $NaHCO_3$ (3 × 10 mL) and brine again (1 × 10 mL), before being dried over anhydrous $MgSO_4$, filtered, and concentrated resulting in a pure white powder (48%). **^{1}H-NMR ($CDCl_3$, 400 MHz):** 1.73 (1H, d, J=2.19 Hz), 2.00 (1H, d, J=2.82 Hz), 2.06 (1H, d, J=0.00 Hz), 3.56 (1H, d, J=0.00 Hz), 3.70 (1H, d, J=0.00 Hz), 5.74 (1H, q, J=4.12 Hz), 6.32 (1H, dd, J=1.94, 16.79 Hz), 6.35 (1H, d, J=1.95 Hz), 6.56 (1H, ddd, J=10.41, 16.81, 0.00 Hz), 6.59 (1H, d, J=10.38 Hz). **^{13}C-NMR ($CDCl_3$, 101 MHz): 28.41, 36.57, 39.08, 41.78, 45.87, 127.09, 128.56, 165.61, 176.11. HRMS-EI:** $[M]^+$ = calculated 302.1994 found 302.1991.

N-[2-(morpholin-4-yl)-2-oxoethyl]prop-2-enamide (5). Compound **9** (0.15 g, 1.6 mmol, 1 eq), morpholine (0,182 mL, 2.11 mmol, 1.3 eq) and NMI (0,453 mL, 5.67 mmol, 3.5 eq) were dissolved in ACN at room temperature and under nitrogen. Then, Chloro-N,N,N′,N′-tetramethylformamidinium hexafluorophosphate (TCHF, 0.544 g, 1.94 mmol, 1.2 eq) was added and the reaction was stirred overnight (TLC: MeOH/DCM, 8/92, completion was not reached). ACN was then evaporated, and the resulting mixture was dissolved in AcOEt and washed with AcOH 5% (3 times), brine (once), saturated $NaHCO_3$ (3 times) and brine again. The resulting aqueous phase was then extracted three times with AcOEt. The resulting yellow oil was then purified by flash chromatography (MeOH/ DCM, gradient 0 to 8% MeOH). **^{1}H-NMR ($CDCl_3$, 300 MHz):** 3.43 (2H, t, J=4.87 Hz), 3.67 (6H, m), 4.12 (2H, d, J=4.14 Hz), 5.66 (1H, dd, J=3.91 Hz), 6.18 (1H, q, J=8.97 Hz), 6.30 (1H, dd, J=6.29 Hz), 6.76 (1H, s). **^{13}C-NMR ($CDCl_3$, 101 MHz):** 41.15, 42.31, 44.81, 66.31, 66.65, 126.88, 130.32, 165.36, 166.58. **HRMS-EI:** M^+ = calculated 198.1004 found 198.1016.

***N*-(carbamoylmethyl)prop-2-enamide (6).** Glycinamide (0.25 g, 2.25 mmol, 1 eq) and K_2CO_3 (0.625 g, 4.5 mmol, 2 eq) were dissolved in MilliQ H_2O (15 mL) then cooled to about 5°C. Then acryloyl chloride in Et_2O (0.2 mL, 2.45 mmol, 1.1 eq) was added very slowly over 25 min. The reaction was warmed up to room

temperature and stirred for 90 min until completion (DCM/MeOH, 95/5). The solvents were evaporated, and acetone (minimal amount) was added to the mixture to precipitate K_2CO_3. After filtration, the acetone solution was used to recrystallize the desired product into white crystals with a yield of 55%. The spectral data match those reported in the literature [44].

2-(prop-2-enamido)acetic acid (9). Glycine t*ert*-butyl ester hydrochloride (2.5 g, 15 mmol, 1 eq) and DIPEA (5.2 mL, 30 mmol, 2 eq) were dissolved in dry DCM under N_2 and cooled to 0°C. Acryloyl chloride (1.4 mL, 17 mmol, 1.1 eq) was added dropwise slowly. The reaction was then stirred at to room temperature until completion (TLC eluant DCM: MeOH 98:2), about 30 min. DCM was evaporated, and the residue was then dissolved in AcOEt (50 mL). This organic layer was washed with AcOH 5% (3 × 50 mL), brine (3 × 50 mL), saturated $NaHCO_3$ (3 × 50 mL) and brine again (3 × 50 mL) before being dried over anhydrous $MgSO_4$, filtered and concentrated. The resulting crude yellow oil was directly dissolved in neat TFA (20 mL) and stirred at room temperature until completion (TLC eluant DCM: MeOH, 98:2, 60 min). TFA was then evaporated and co-evaporated with DCM 5 times. The resulting oil was dissolved in cold Et_2O, resulting in formation of a white powder with an overall yield of 50%. The characterization matches the literature [45].

***N*-(Adamantanecarbonyl)piperazine hydrochloride (10)** was synthesized as previously described [34].

2-Amino-1-[4-(1-adamantanecarbonyl)-1-piperazinyl]ethenone hydrochloride (11) was synthesized as previously described [34].

Benzyl N-[2-oxo-2-(piperazin-1-yl)ethyl]carbamate hydrochloride (12). Cbz-Gly-OH (1 g, 4.8 mmol, 1.1 eq), Boc-piperazine (0.81 g, 4.4 mmol, 1 eq), HATU (2.65 g, 7 mmol, 1.6 eq) and DIPEA (2.3 mL, 13.2 mmol, 3 eq) were dissolved in dry DCM (20 mL) and the reaction was monitored by TLC (DCM/MeOH, 98/2) and stirred at room temperature overnight. The solvent was then evaporated, and the resulting orange oil was dissolved in EtOAc (50 mL). The resulting organic phase was washed with HOAc 5% (3 × 50 mL), followed by one wash with brine, then saturated $NaHCO_3$ (3 × 50 mL) and brine again (3 × 50 mL). The organic layer was then dried over anhydrous $MgSO_4$, filtered and concentrated. The resulting orange oil was directly dissolved in a mixture of HCl (4 M)/dioxane and DCM (1:1, 20 mL) and the reaction was stirred at room temperature until completion (followed by TLC, DCM/MeOH, 98/2, 3.5 h). The solvents were then evaporated, and the resulting powder was washed with cold Et_2O. The desired product was obtained

in an overall yield of 85% as a slightly pink colored powder. The characterization matches the literature [46].

Benzyl N-[2-(4-acetylpiperazin-1-yl)-2-oxethyl]carbamate (13). Compound **12** (0.5 g, 1.6 mmol, 1 eq) was suspended in DCM under N_2 atmosphere and TEA (0.67 mL, 4.8 mmol, 3 eq) and the mixture was cooled at 0°C. Acetyl chloride (0.17 mL, 2.4 mmol, 1.5 eq) was added slowly over 15 min. The reaction was then warmed up to room temperature and stirred until completion, about 6 h (TLC: DCM/MeOH, 95/5). The DCM was then evaporated, and the resulting oil was dissolved in Me-THF (20 mL) and washed with AcOH 5% (3 × 20 mL) and brine (3 × 20 ml). TLC showed that most of the compound stays in the aqueous layer, which was then re-extracted with Me-THF (3 × 20 mL) and AcOEt (3 × 20 mL). The desired product was then directly used to prepare compound **3.**

2. NMR Spectra

Inhibitor ***2***

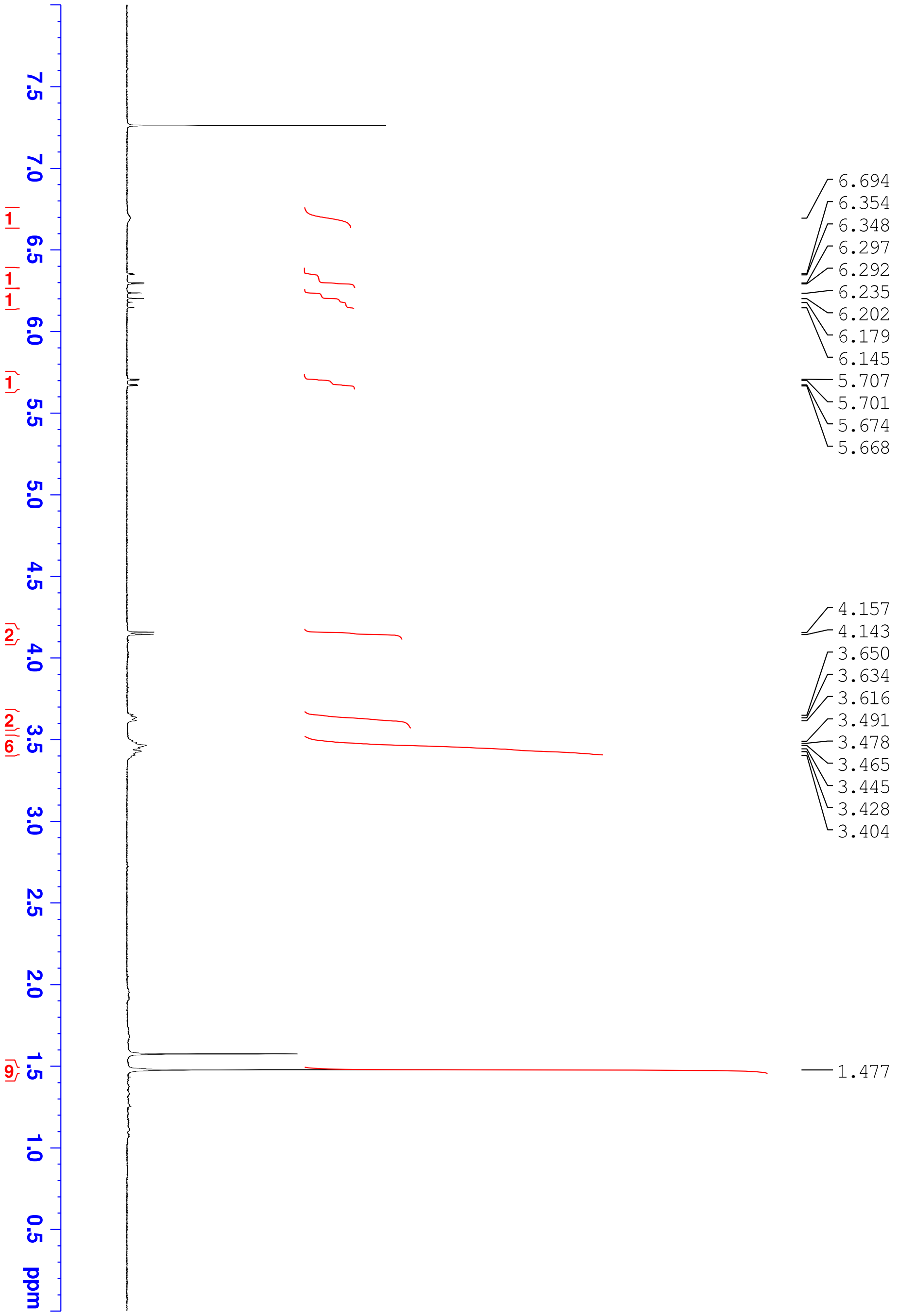

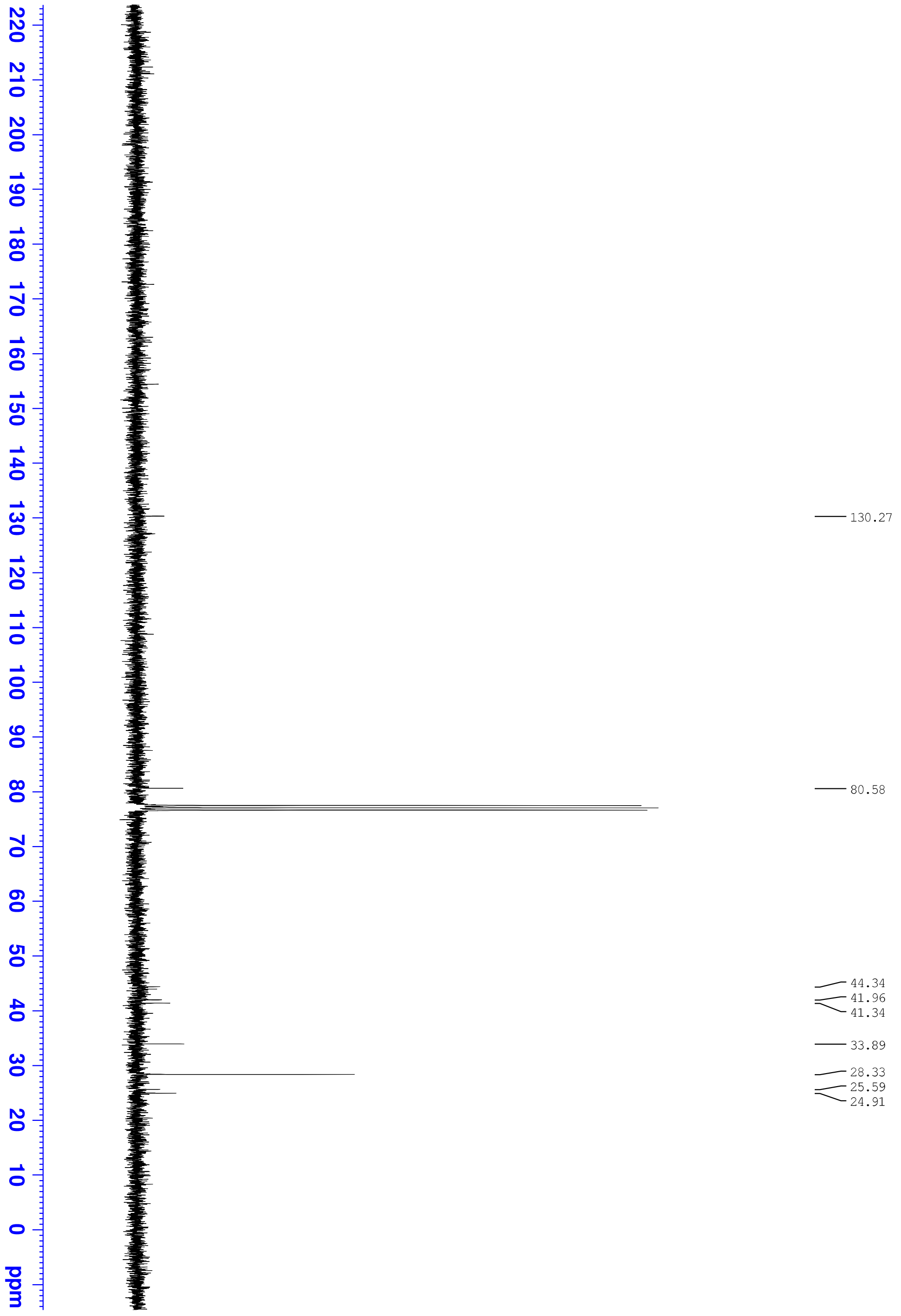
130.27
80.58
44.34
41.96
41.34
33.89
28.33
25.59
24.91
ppm

*Inhibitor **3***

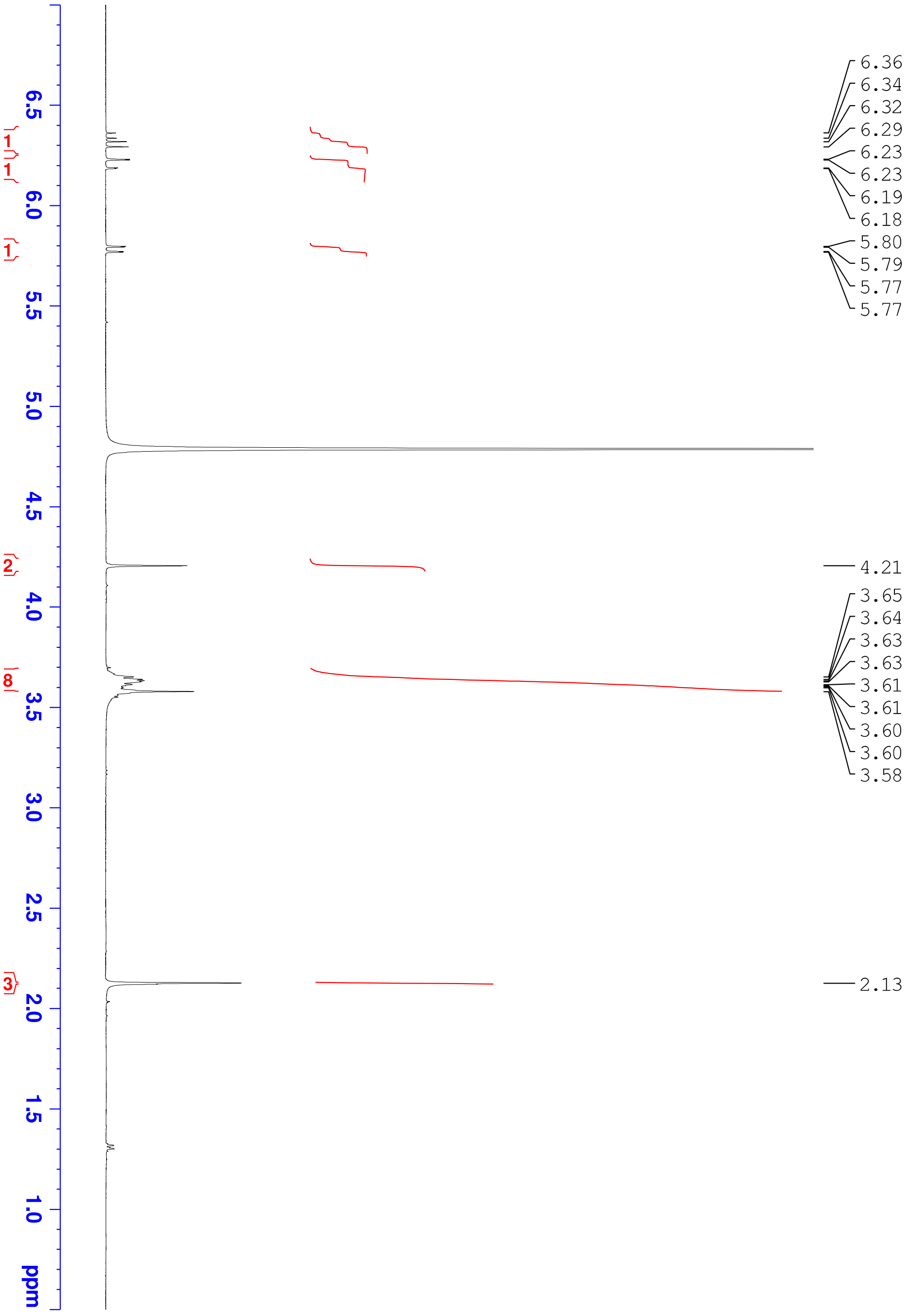
6.36
6.34
6.32
6.29
6.23
6.23
6.19
6.18
5.80
5.79
5.77
5.77
4.21
3.65
3.64
3.63
3.63
3.61
3.61
3.60
3.60
3.58
2.13
6.5
6.0
5.5
5.0
4.5
4.0
3.5
3.0
2.5
2.0
1.5
1.0
ppm

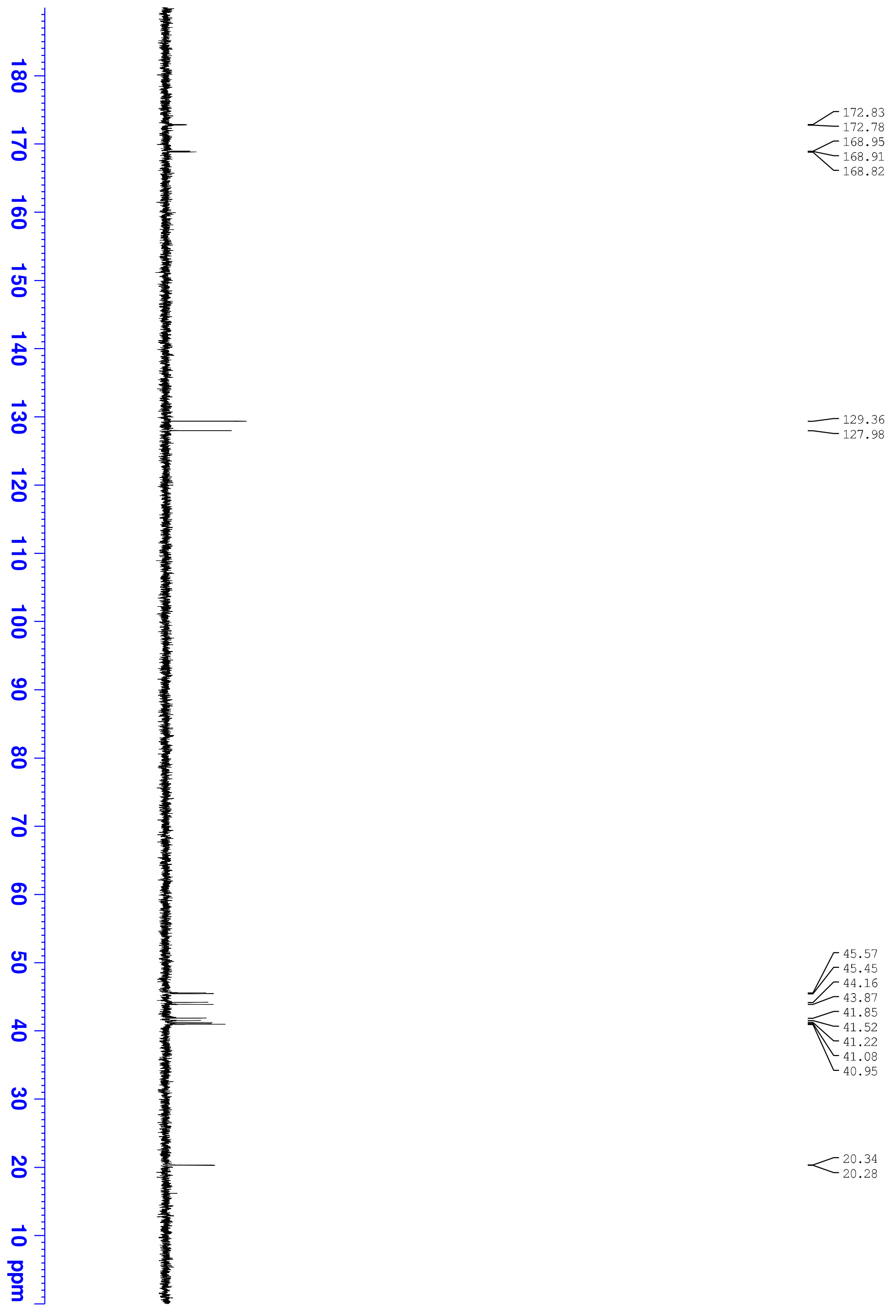
172.83
172.78
168.95
168.91
168.82
129.36
127.98
45.57
45.45
44.16
43.87
41.85
41.52
41.22
41.08
40.95
20.34
20.28
180 170 160 150 140 130 120 110 100 90 80 70 60 50 40 30 20 10 ppm

Inhibitor ***4***

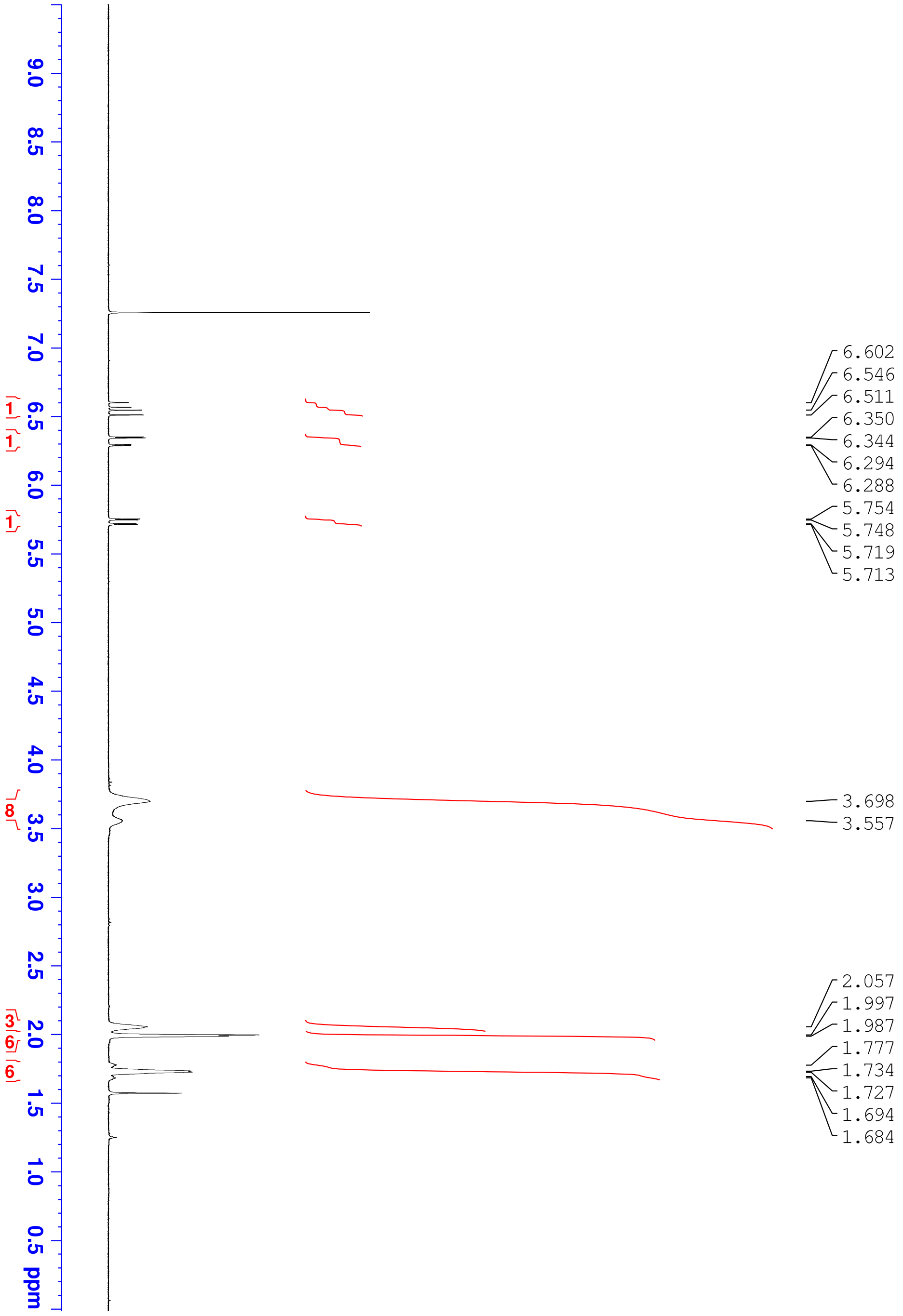
6.602
6.546
6.511
6.350
6.344
6.294
6.288
5.754
5.748
5.719
5.713
3.698
3.557
2.057
1.997
1.987
1.777
1.734
1.727
1.694
1.684
9.0
8.5
8.0
7.5
7.0
6.5
6.0
5.5
5.0
4.5
4.0
3.5
3.0
2.5
2.0
1.5
1.0
0.5 ppm
1
1
1
8
3
6
6

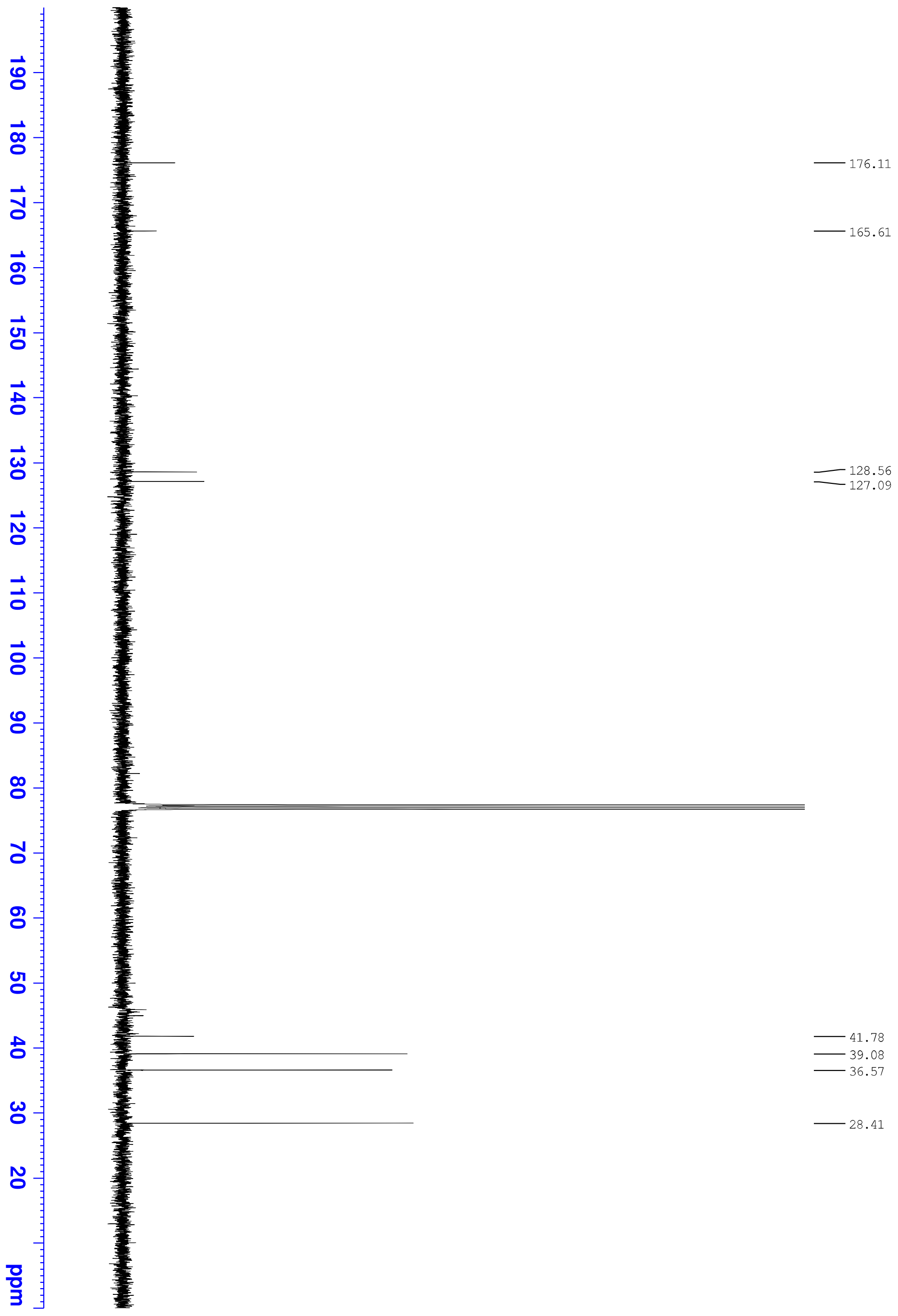
176.11
165.61
128.56
127.09
41.78
39.08
36.57
28.41
190
180
170
160
150
140
130
120
110
100
90
80
70
60
50
40
30
20
ppm

Inhibitor ***5***

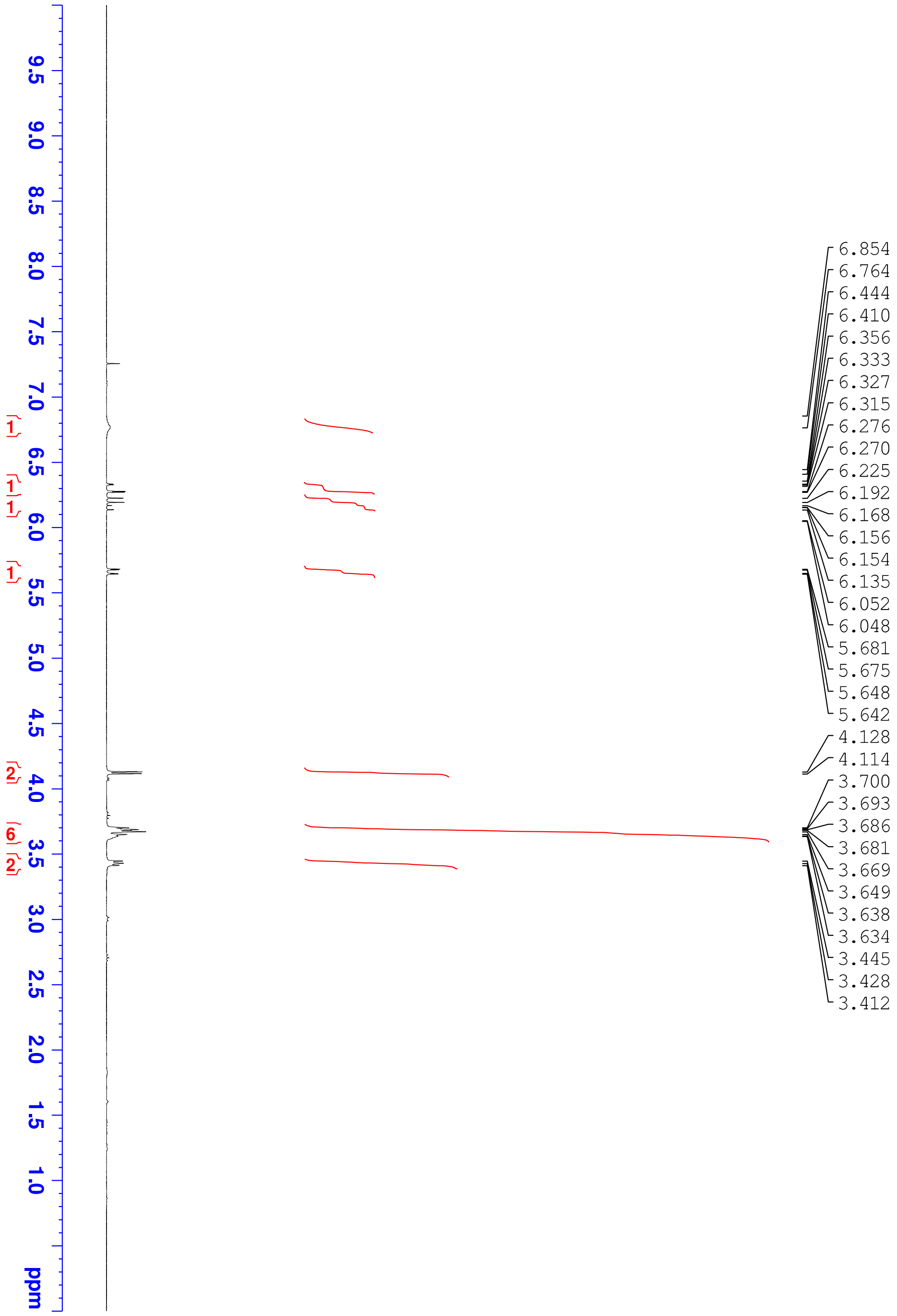
6.854
6.764
6.444
6.410
6.356
6.333
6.327
6.315
6.276
6.270
6.225
6.192
6.168
6.156
6.154
6.135
6.052
6.048
5.681
5.675
5.648
5.642
4.128
4.114
3.700
3.693
3.686
3.681
3.669
3.649
3.638
3.634
3.445
3.428
3.412
ppm

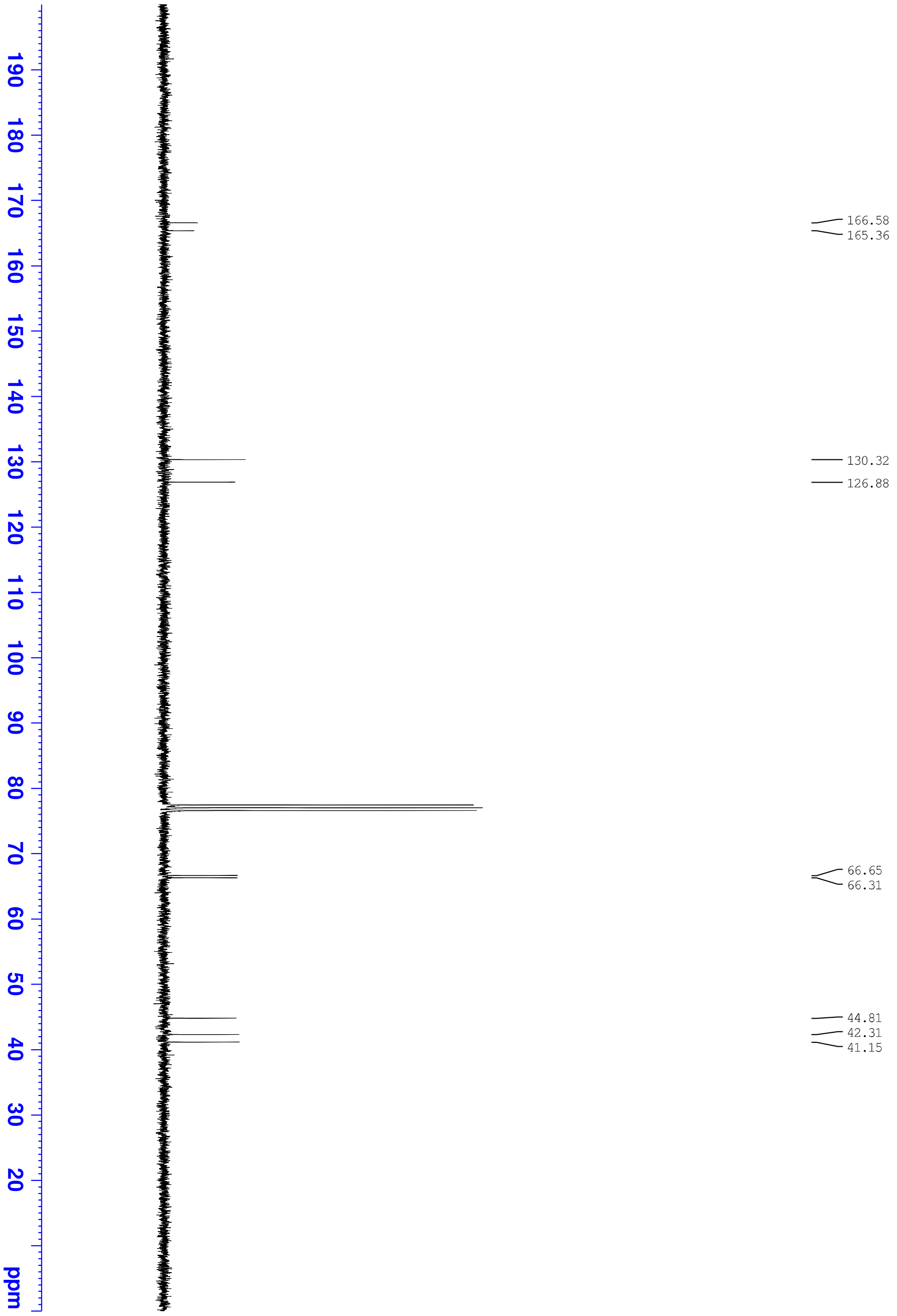
166.58
165.36
130.32
126.88
66.65
66.31
44.81
42.31
41.15
190
180
170
160
150
140
130
120
110
100
90
80
70
60
50
40
30
20
ppm

3. HPLC data

The purity of the final inhibitors was determined by high performance liquid chromatography (HPLC, Gilson-Mandel GXP271). UV detection was performed at 214 and 254 nm (Phenomenex Luna, 150 mm × 4.6 mm) according to two methods:
Method one :10-70% acetonitrile in H_2O + 0.1% TFA, 1mL/min
Method two: 2-25% acetonitrile in H_2O + 0.1% TFA, 1mL/min

Table S1: HPLC purity data for synthesized inhibitor 1 to 6

Compound	Retention time (min)	Relative purity (%)
**1**[*]	16.1	96.3
**2** [a]	10.60	96.8
**3** [b]	2.092	98.8
**4** [a]	15.70	96.1
**5** [b]	2.26	97.1
**6** [b]	1.50	99.4

* From previous study (*RSC Med. Chem.*, **2022**, *13*, 413-428)
[a] method 1
[b] method 2

Inhibitor ***1***

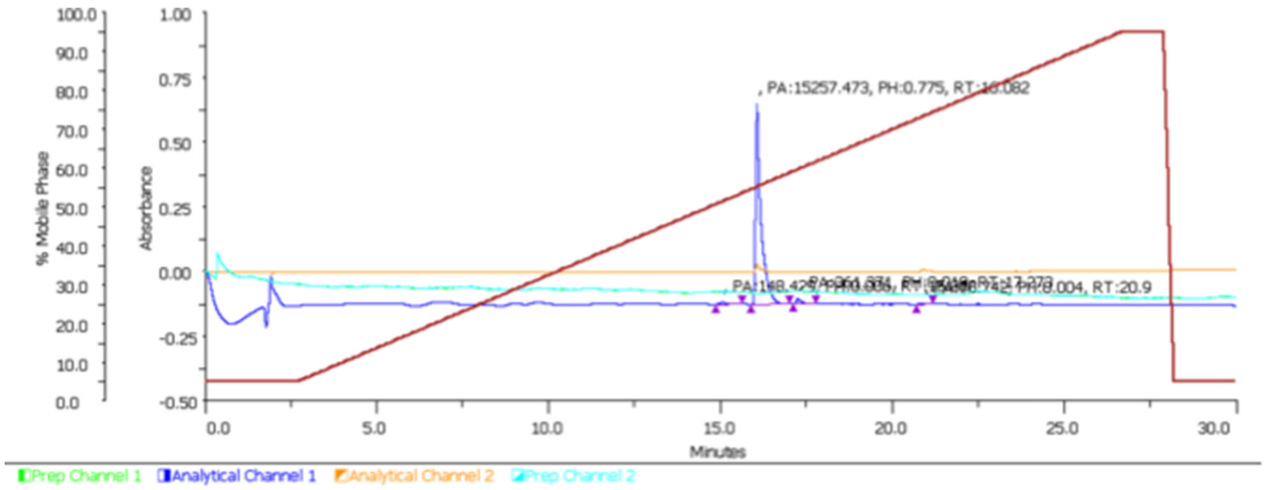

Sample Table

Injection Number	Peak Name	Retention Time (min)	Area (mAUmin x100)	Height (AU)	Sample Name	Sample Location	Fraction Site(s)	Area %	
4	1	16.082	15257.4729	0.775	AR2-82	Sample Zone->153		96.298	
4	2	17.273	361.3712	0.019	AR2-82	Sample Zone->153		2.281	
4	3	20.9	76.7417	0.004	AR2-82	Sample Zone->153		0.484	
4	4	15.068	148.4287	0.006	AR2-82	Sample Zone->153		0.937	

Inhibitor **2**

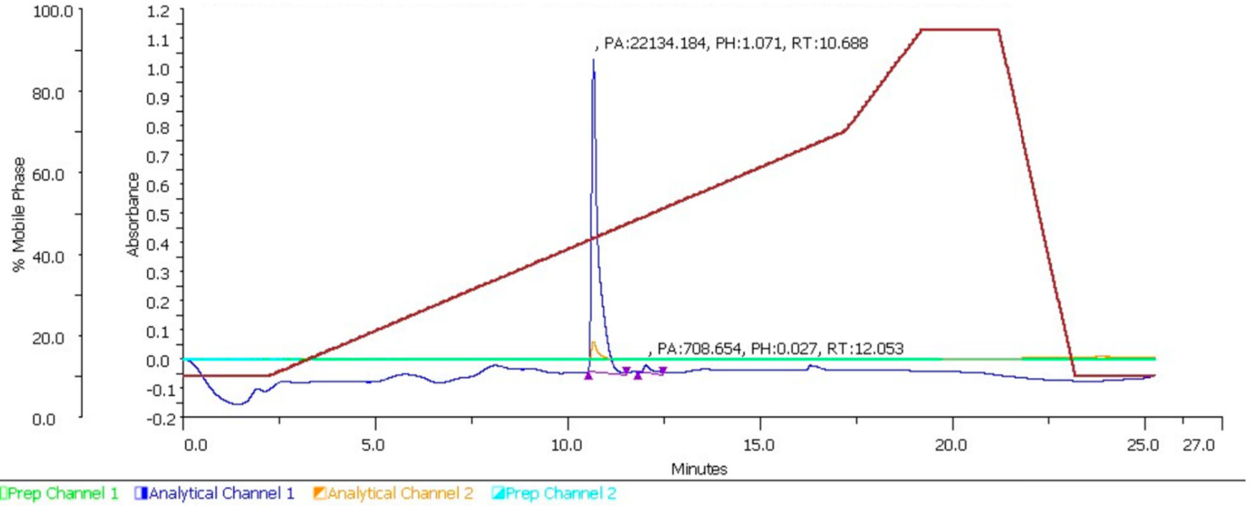

Sample Table

Injection Number	Peak Name	Retention Time (min)	Area (mAUmin x100)	Height (AU)	Sample Name	Sample Location	Fraction Site(s)	Area %	
2	1	10.688	22134.1845	1.071	boc-pip-PN146	Sample Zone->152		96.898	
2	2	12.053	708.6542	0.027	boc-pip-PN146	Sample Zone->152		3.102	

Inhibitor 3

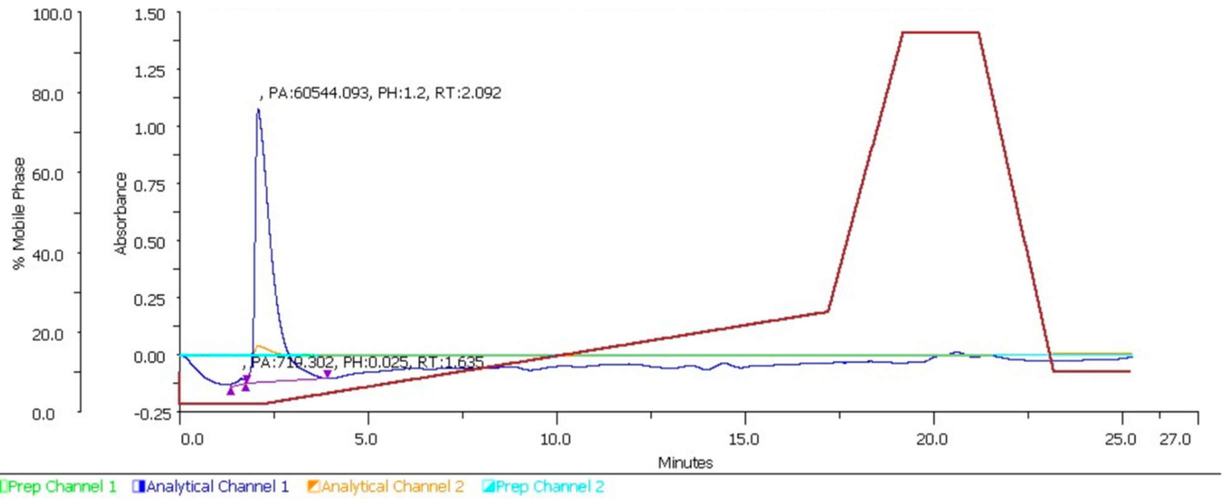

Sample Table

Injection Number	Peak Name	Retention Time (min)	Area (mAUmin x100)	Height (AU)	Sample Name	Sample Location	Fraction Site(s)	Area %	
2	1	1.635	719.3022	0.025	PN173-C18pull	Sample Zone->156		1.174	
2	2	2.092	60544.0934	1.2	PN173-C18pull	Sample Zone->156		98.826	

*Inhibitor **4***

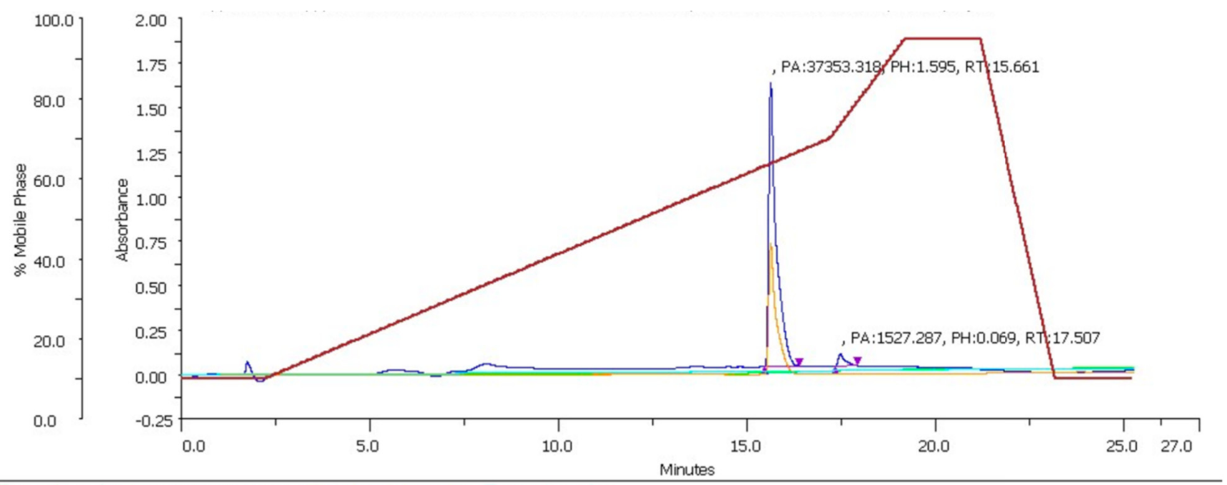

Sample Table

Injection Number	Peak Name	Retention Time (min)	Area (mAUmin x100)	Height (AU)	Sample Name	Sample Location	Fraction Site(s)	Area %	
1	1	15.661	37353.3177	1.595	Admfinal-PN111	Sample Zone->156		96.072	
1	2	17.507	1527.2873	0.069	Admfinal-PN111	Sample Zone->156		3.928	

Inhibitor **5**

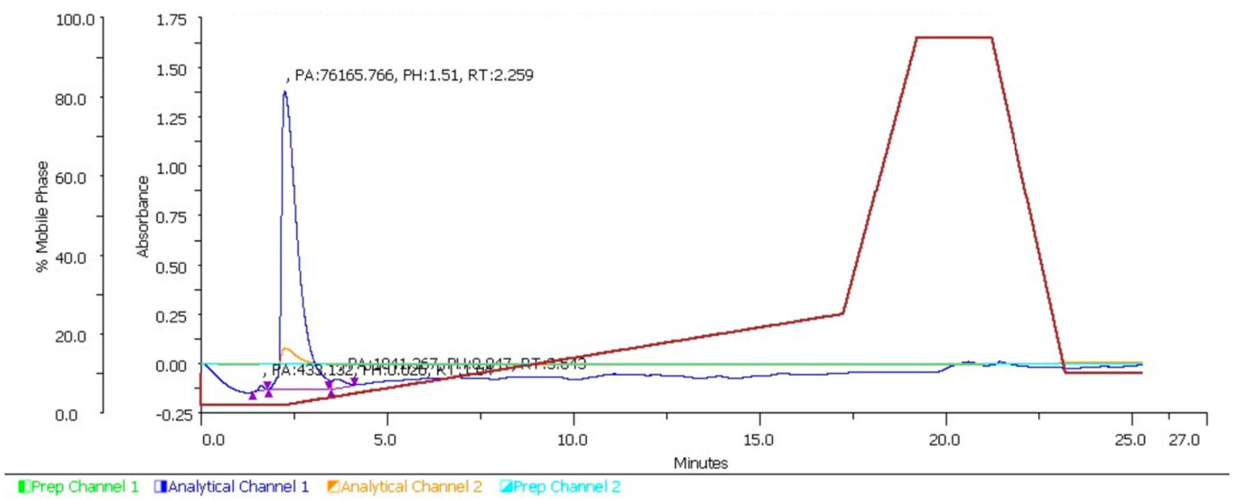

Sample Table

Injection Number	Peak Name	Retention Time (min)	Area (mAUmin x100)	Height (AU)	Sample Name	Sample Location	Fraction Site(s)	Area %	
2	1	1.64	433.1321	0.026	PN-150	Sample Zone->162		0.552	
2	2	2.259	76165.766	1.51	PN-150	Sample Zone->162		97.1	
2	3	3.643	1841.3668	0.047	PN-150	Sample Zone->162		2.347	

Inhibitor **6**

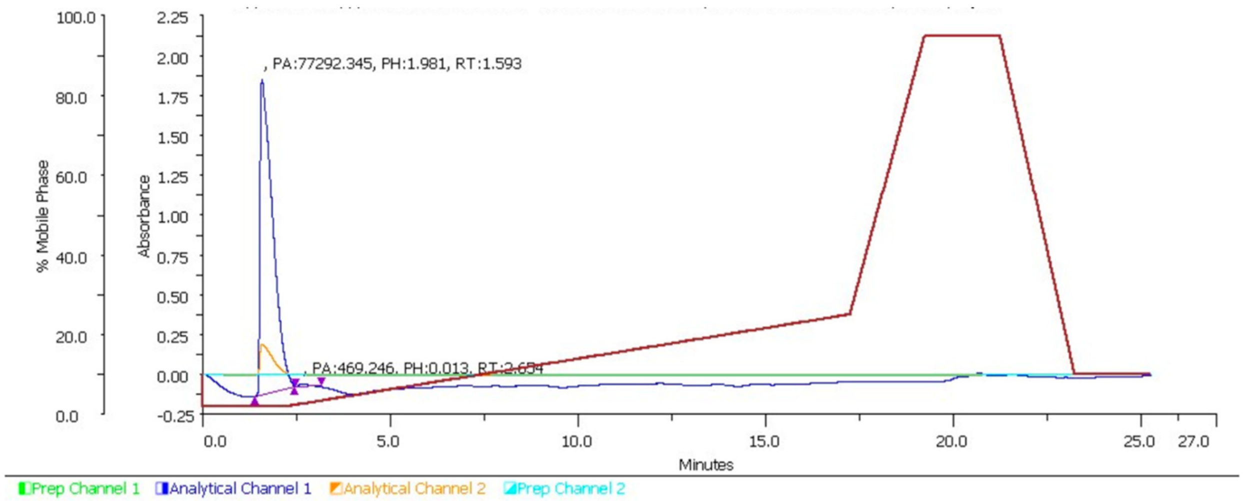

Sample Table

Injection Number	Peak Name	Retention Time (min)	Area (mAUmin x100)	Height (AU)	Sample Name	Sample Location	Fraction Site(s)	Area %	
2	1	1.593	77292.3449	1.981	PN-174	Sample Zone->163		99.397	
2	2	2.654	469.2465	0.013	PN-174	Sample Zone->163		0.603	

4. Native PAGE original stained gels

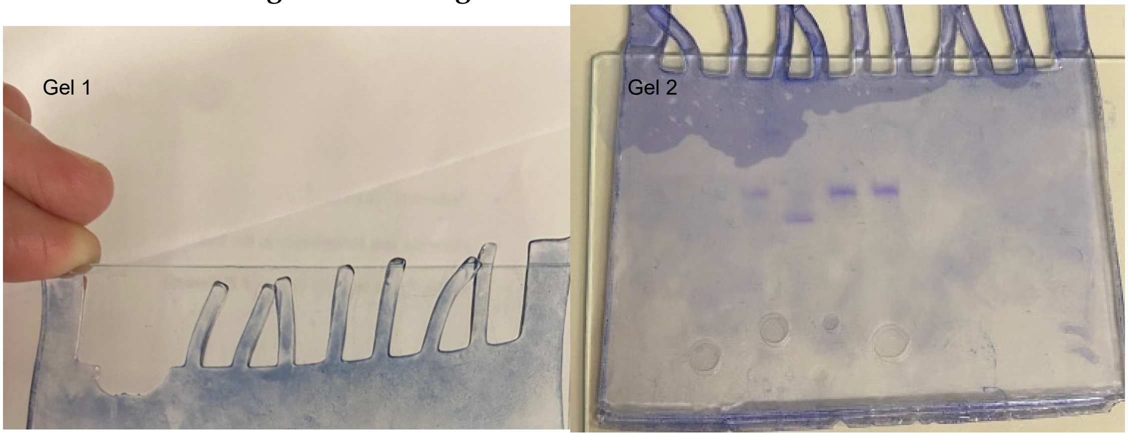

Figure S1: Original images of the full-length native PAGE gels. Gel 1: Controls (lanes 1 to 3) + inhibitor VA4, 1, 5,7, 8 and Iodoacetamide (lanes 4 to 9). Gel 2: Controls (lanes 3 and 4) and iodoacetamide (5 eq and 10 eq, lanes 5 and 6).

5.Capillary electrophoresis full electropherograms

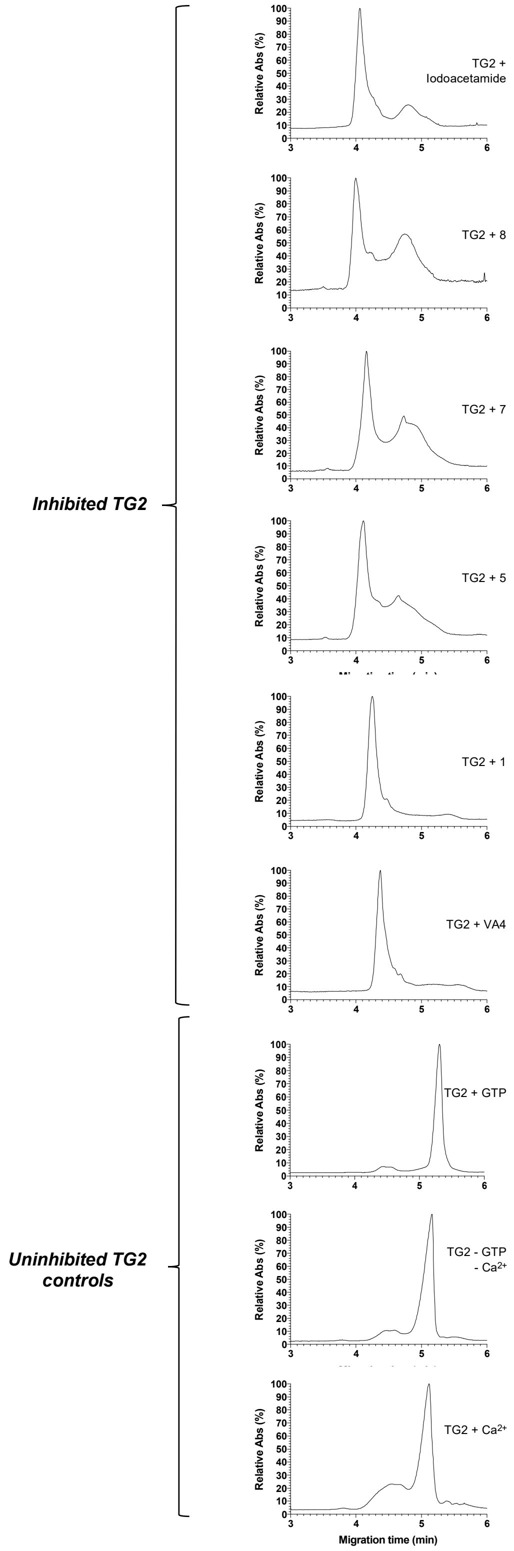

Figure S2: KCE electropherograms of comparative analysis of all analytes.

Control 1: TG2 + Ca^{2+}

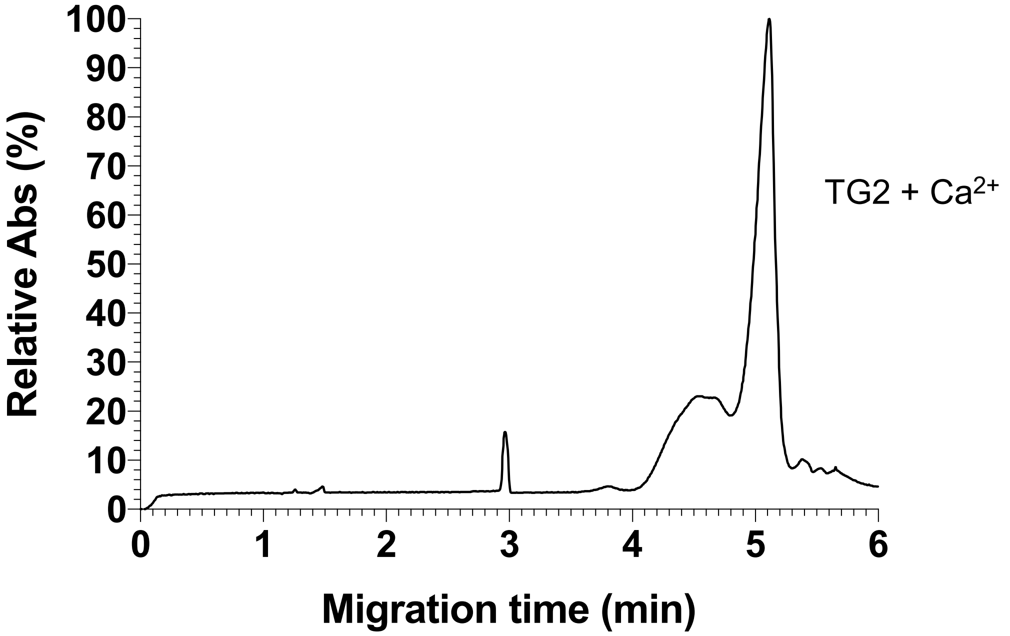

Control 2: TG2 -GTP and -Ca^{2+}

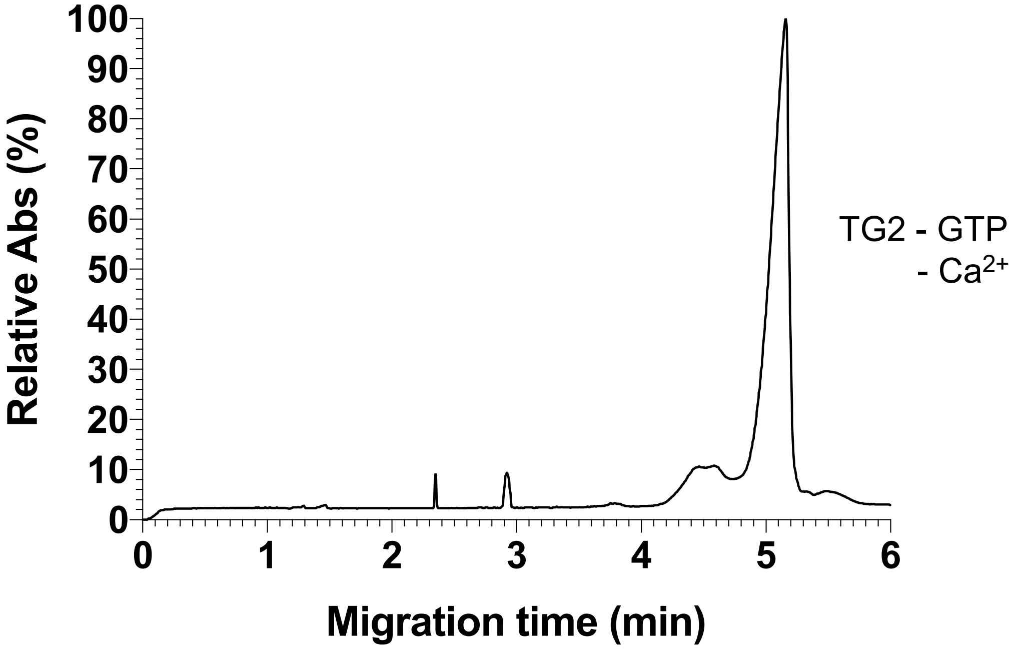

Control 3: TG2 + GTP

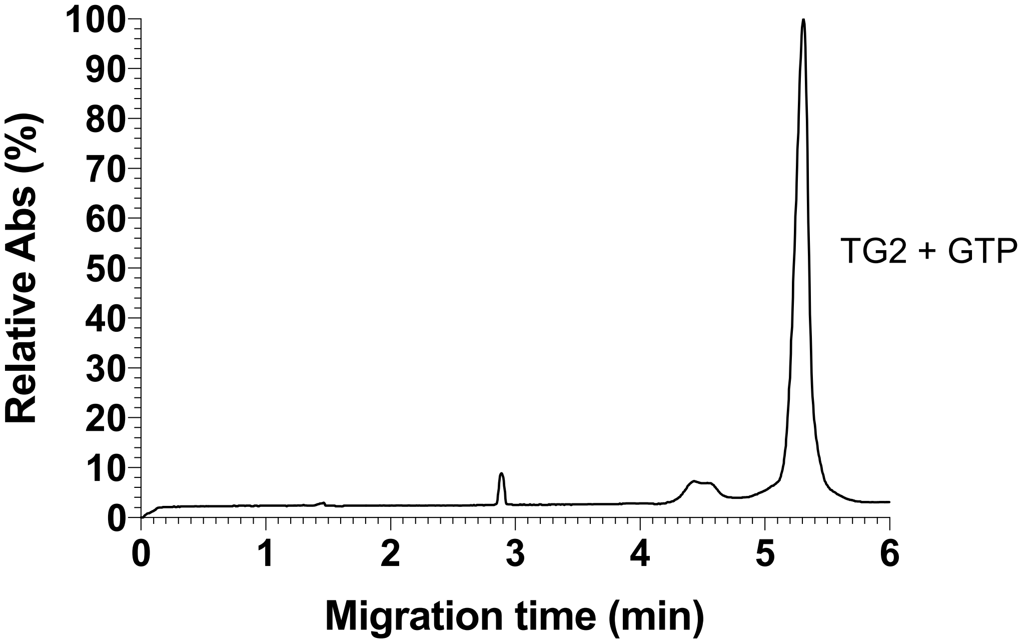

TG2 + inhibitor ***VA4***

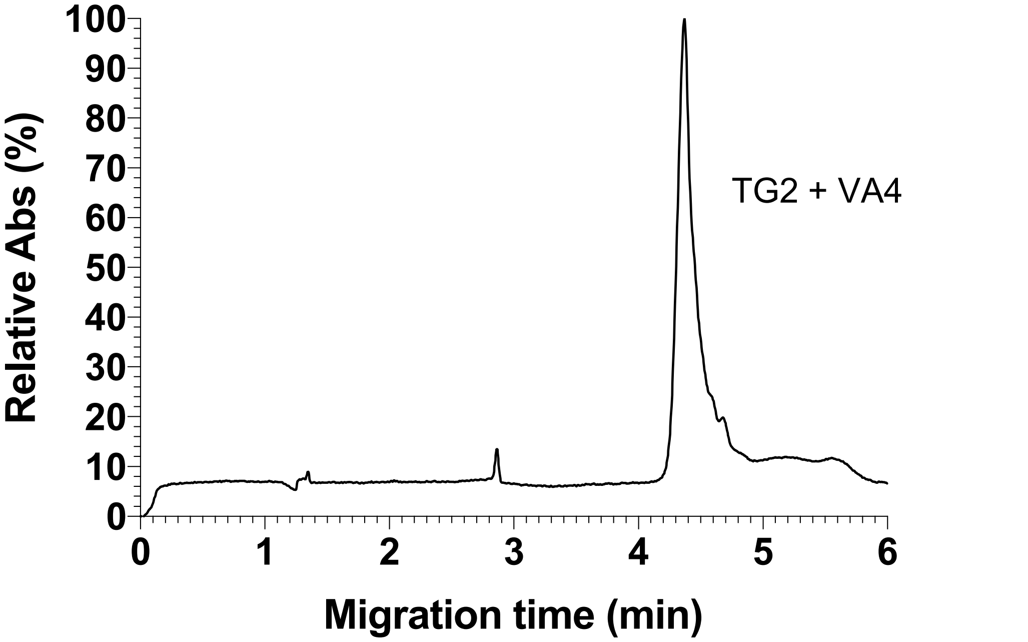

*TG2 + inhibitor **1***

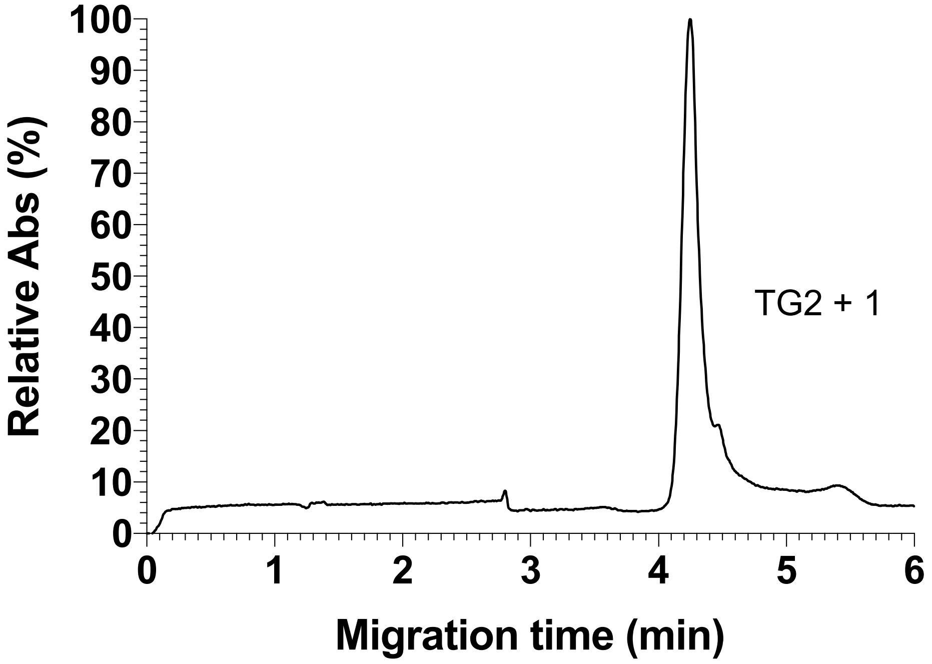

*TG2 + inhibitor **5***

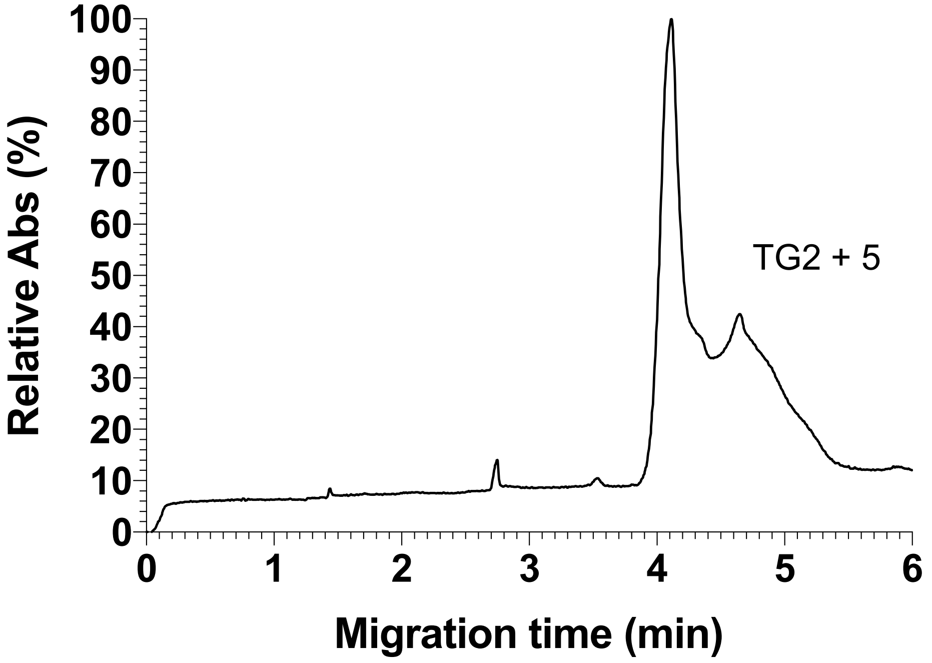

TG2 + inhibitor 7

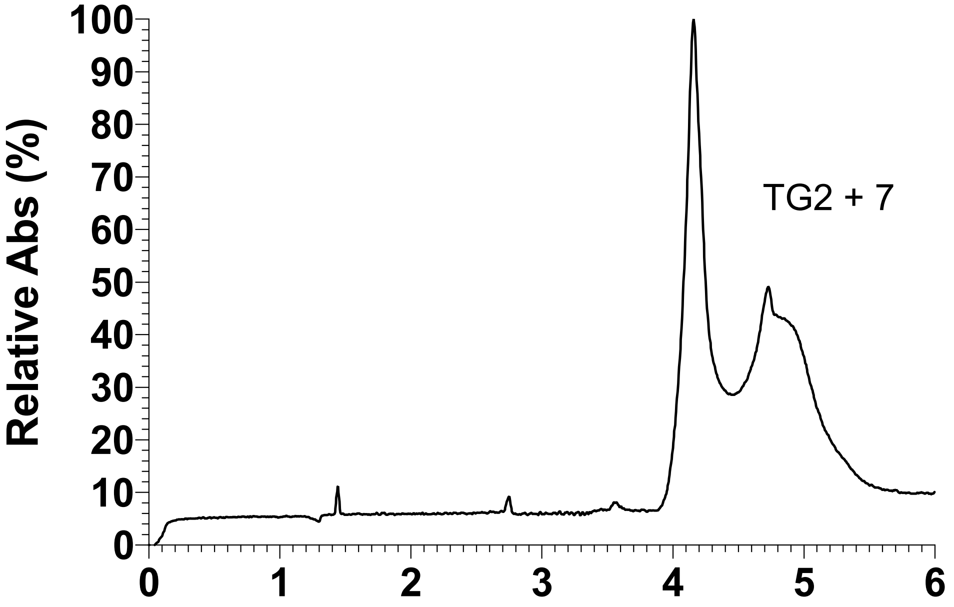

TG2 + inhibitor 8

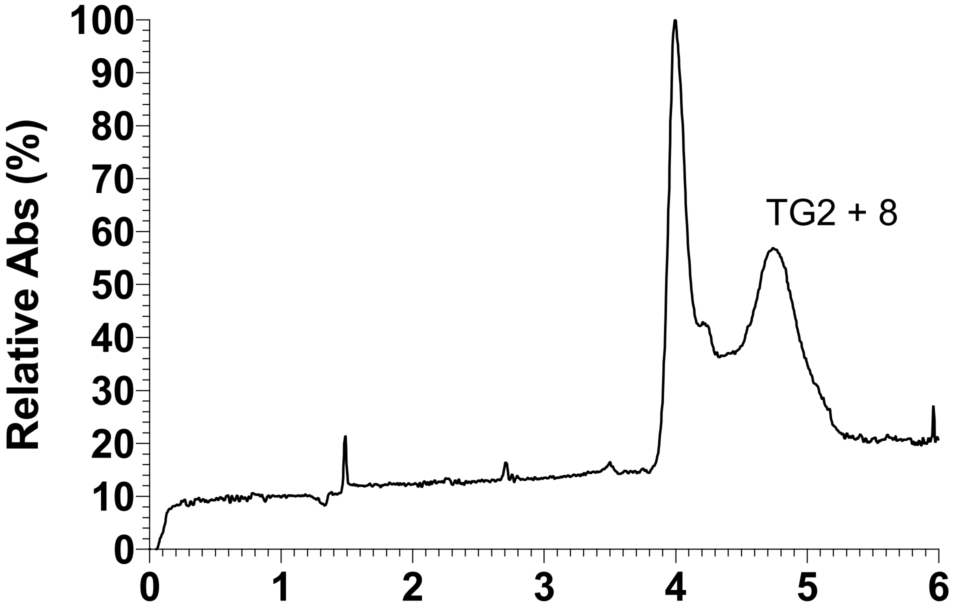

TG2 + Iodoacetamide

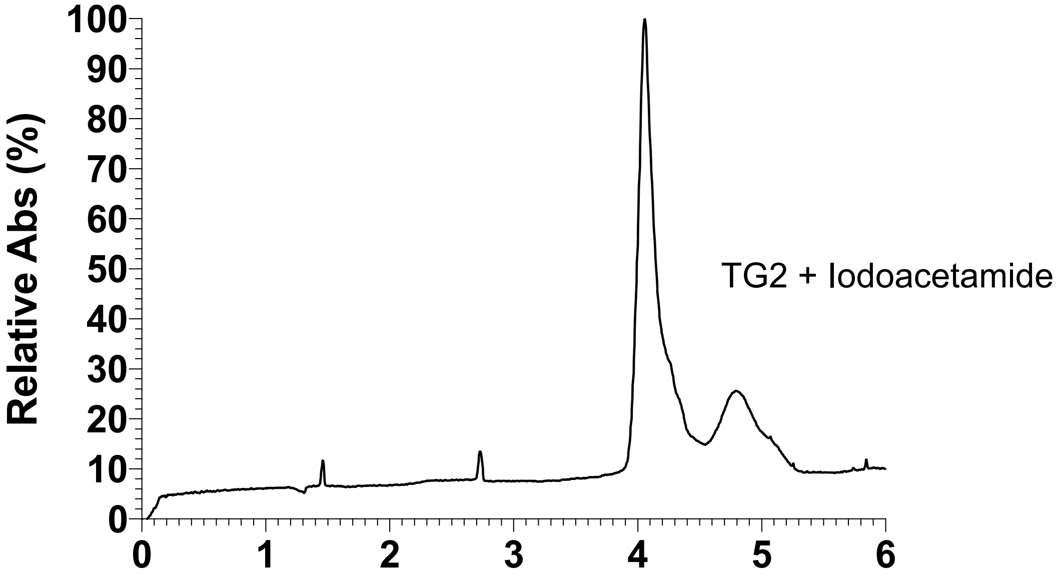

6. Kinetic data fitting

Inhibitor ***1****

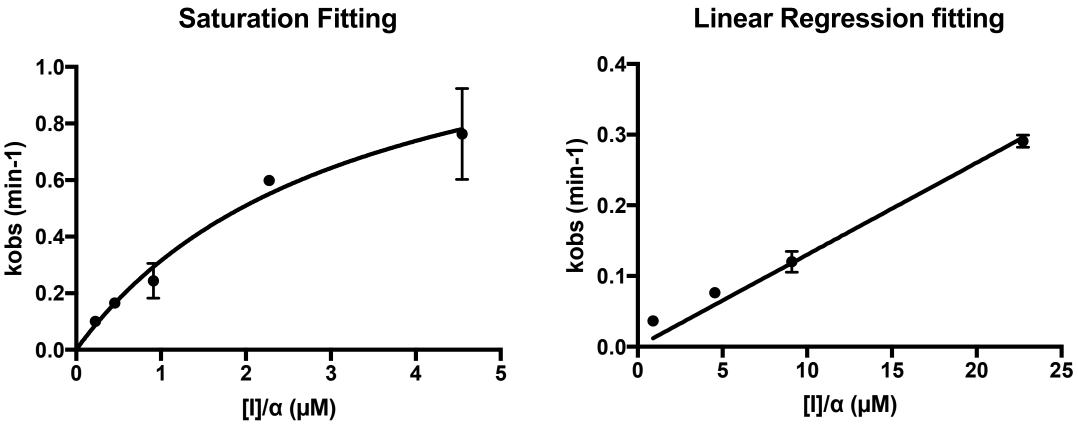

* Previously reported (*RSC Med. Chem.*, **2022**, *13*, 413-428)

Inhibitor **2**

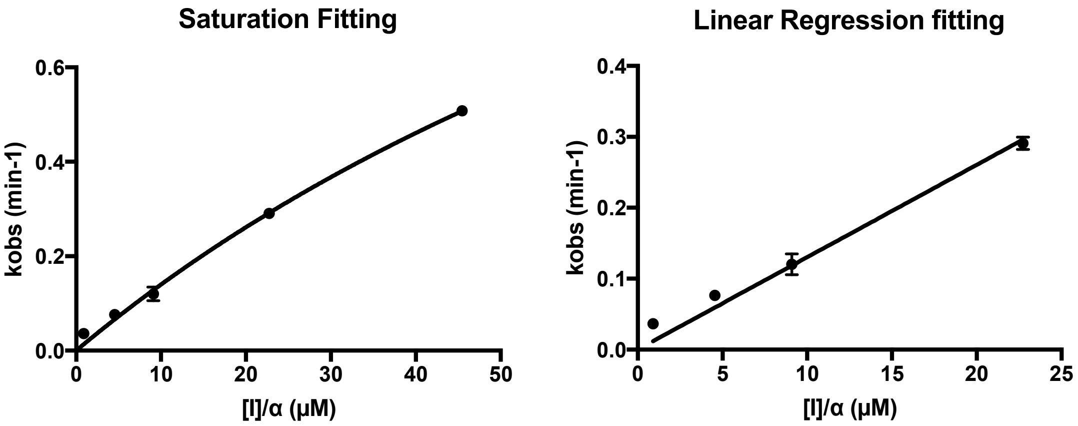

*Inhibitor **3***

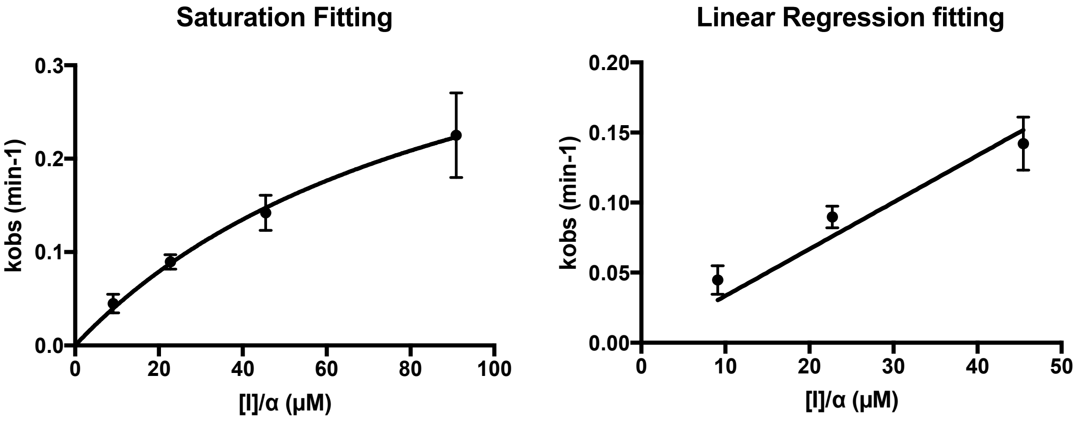

*Inhibitor **4***

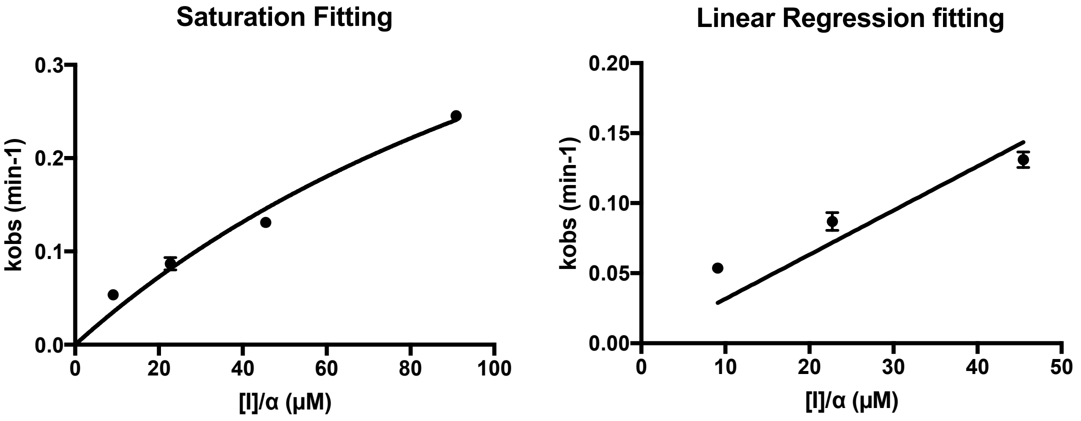

*Inhibitor **5***

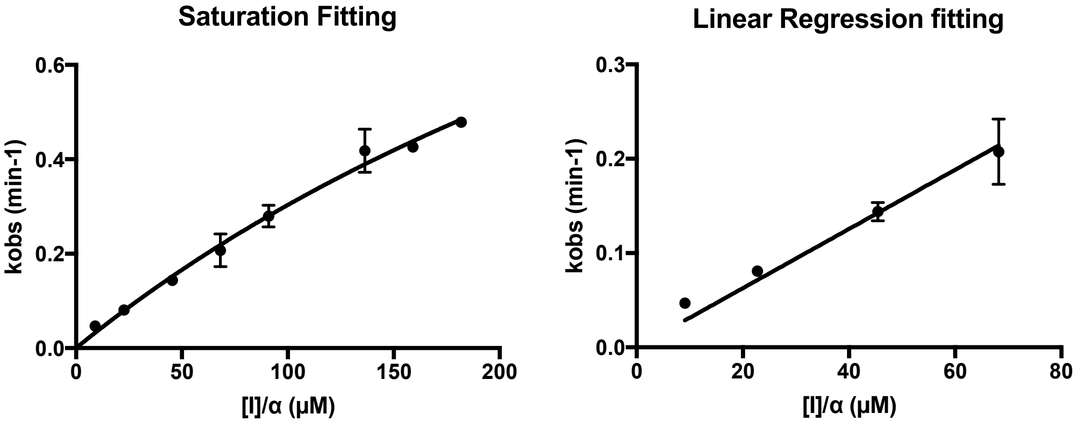

*Inhibitor **6***

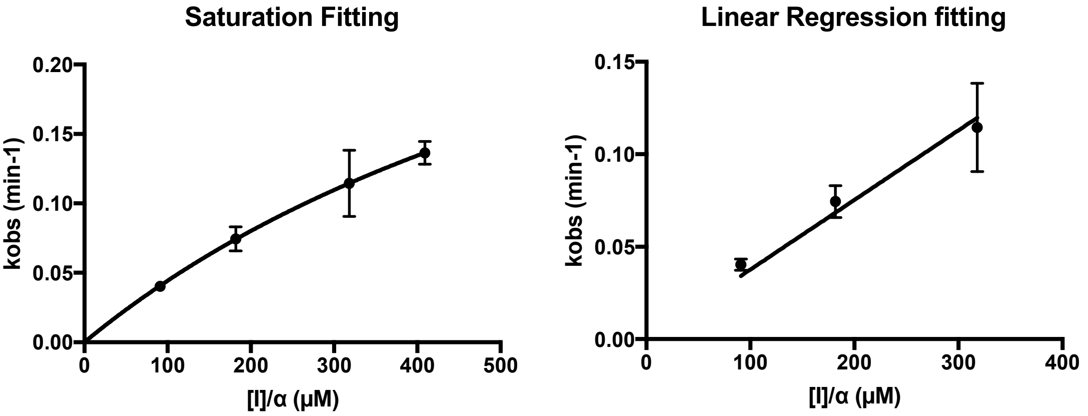

Inhibitor 7

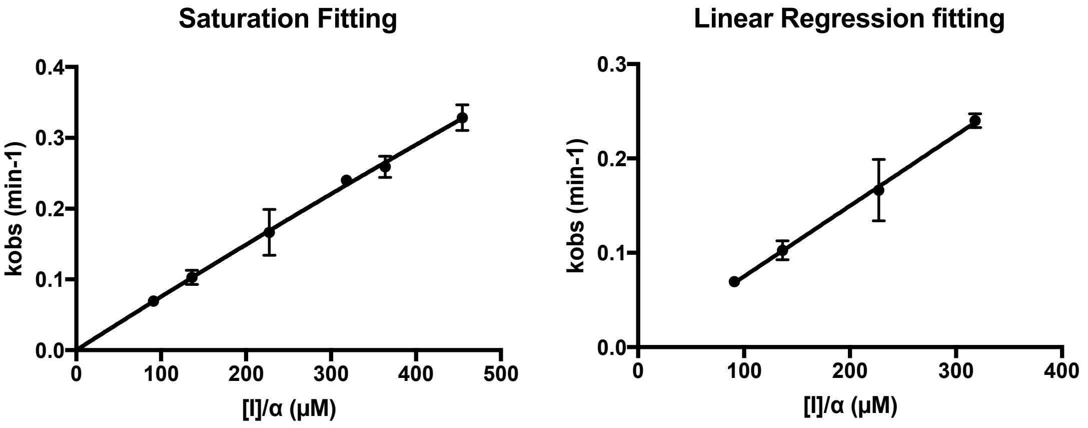

Inhibitor 8

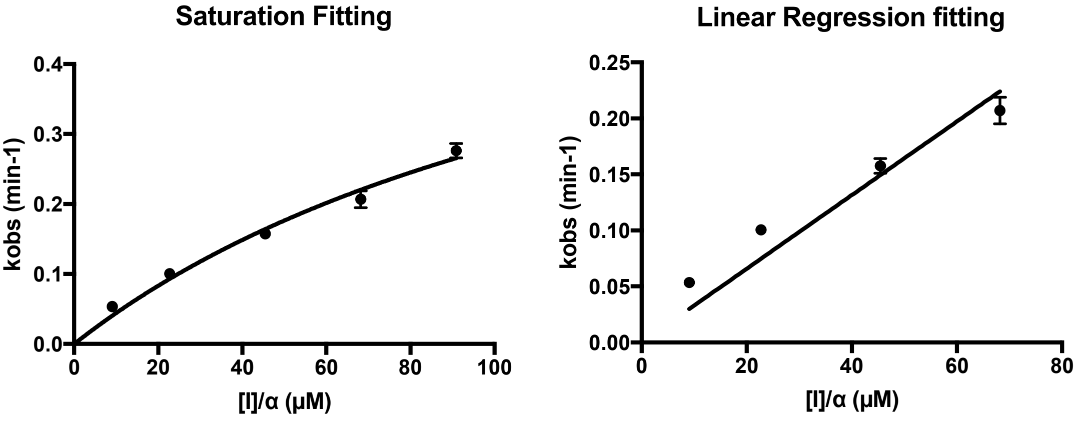

References

34. Rangaswamy, A.M.M.; Navals, P.; Gates, E.W.J.; Shad, S.; Watt, S.K.I.; Keillor, J.W. Structure–Activity Relationships of Hydrophobic Alkyl Acrylamides as Tissue Transglutaminase Inhibitors. *RSC Med Chem* **2022**, *13*, 413–428, doi:10.1039/D1MD00382H.
44. Glatzel, S.; Badi, N.; Päch, M.; Laschewsky, A.; Lutz, J.-F. Well-Defined Synthetic Polymers with a Protein-like Gelation Behavior in Water. *Chemical Communications* **2010**, *46*, 4517, doi:10.1039/c0cc00038h.
45. Auernheimer, J.; Dahmen, C.; Hersel, U.; Bausch, A.; Kessler, H. Photoswitched Cell Adhesion on Surfaces with RGD Peptides. *J Am Chem Soc* **2005**, *127*, 16107–16110, doi:10.1021/ja053648q.
46. Agha, K.A.; Abo-Dya, N.E.; Ibrahim, T.S.; Abdel-Aal, E.H.; Abdel-Samii, Z.K. N-Acylbenzotriazole: Convenient Approach for Protecting Group-Free Monoacylation of Symmetric Diamines. *Monatshefte für Chemie - Chemical Monthly* **2020**, *151*, 589–598, doi:10.1007/s00706-020-02579-5.
